# Furanonaphthoquinones, Diterpenes, and Flavonoids from Sweet Marjoram and Investigation of Antimicrobial, Bacterial Efflux, and Biofilm Formation Inhibitory Activities

**DOI:** 10.1021/acsomega.3c03982

**Published:** 2023-09-14

**Authors:** Tasneem Sultan Abu Ghazal, Katalin Veres, Lívia Vidács, Nikoletta Szemerédi, Gabriella Spengler, Róbert Berkecz, Judit Hohmann

**Affiliations:** † Institute of Pharmacognosy, University of Szeged, Szeged H-6720, Hungary; ‡ Department of Medical Microbiology, Albert Szent-Györgyi Health Center and Albert Szent-Györgyi Medical School, University of Szeged, Szeged H-6720, Hungary; § Institute of Pharmaceutical Analysis, University of Szeged, 6720 Szeged, Hungary; ∥ Interdisciplinary Centre for Natural Products, University of Szeged, Szeged H-6720, Hungary; ⊥ ELKH-USZ Biologically Active Natural Products Research Group, University of Szeged, Szeged H-6720, Hungary

## Abstract

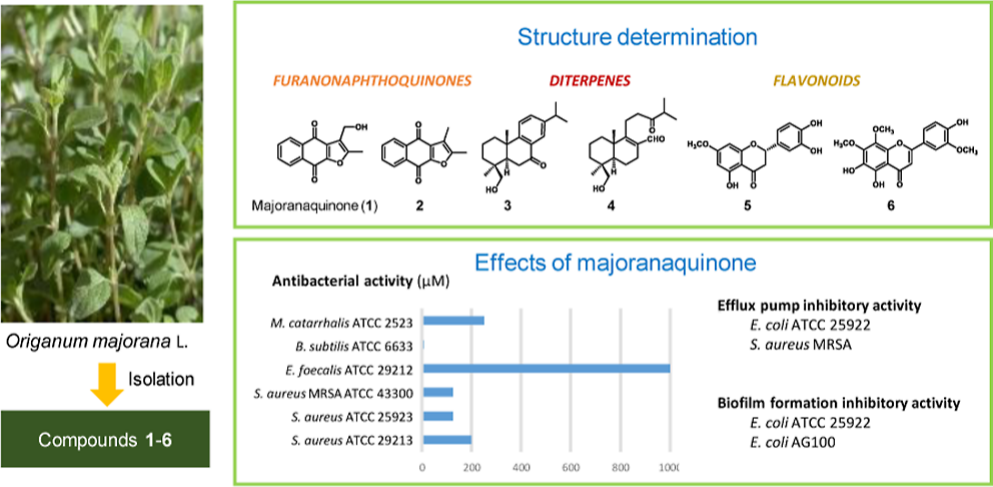

The chloroform extract of *Origanum majorana* exhibited high antibacterial and antifungal activities against 12 bacterial and 4 fungal strains; therefore, it was subjected to bioassay-guided isolation to afford six compounds (**1**–**6**). The structures were determined via one- and two-dimensional nuclear magnetic spectroscopy and high-resolution electrospray ionization mass spectrometry experiments. The compounds were identified as furanonaphthoquinones [majoranaquinone (**1**), 2,3-dimethylnaphtho[2,3-*b*]furan-4,9-dione (**2**)], diterpenes [19-hydroxyabieta-8,11,13-trien-7-one (**3**), 13,14-seco-13-oxo-19-hydroxyabieta-8-en-14-al (**4**)], and flavonoids [sterubin (**5**) and majoranin (**6**)]. Compounds **1** and **2** were first obtained from a natural source and compounds **3** and **4** were previously undescribed. Majoranaquinone (**1**) exhibited a high antibacterial effect against 4 *Staphylococcus*, 1 *Moraxella*, and 1 *Enterococcus* strains (MIC values between 7.8 μM and 1 mM). In the efflux pump inhibition assay, majoranaquinone (**1**) showed substantial activity in *Escherichia coli* ATCC 25922 strain. Furthermore, **1** was found to be an effective biofilm formation inhibitor on *E. coli* ATCC 25922 and *E. coli* K-12 AG100 bacteria. Our findings proved that bioactivities of majoranaquinone (**1**) significantly exceed those of the essential oil constituents; therefore, it should also be considered when assessing the antimicrobial effects of *O. majorana*.

## Introduction

Natural products and their preparations play a continuous and increasing role in human and veterinary medicine, agriculture, food and cosmetics industries, and in more and more other fields. The therapeutic value of plants has been known for a long time, so they are used in home and clinical applications to treat many diseases. Despite the progress of modern medicine that can be observed worldwide, herbal medicines in the form of crude or purified extracts are still used to treat or prevent many pathological conditions. Historically, natural products have played a key role in drug discovery, particularly for infectious diseases and cancer. In addition to structural complexity, great chemical diversity, and remarkable biological activity, their importance is also that they are mostly linked to renewable sources, which makes them valuable in terms of the circular economy.^1,2^

*Origanum majorana* L., (Lamiaceae) commonly known as marjoram or sweet marjoram, is a widely used medicinal and aromatic plant. The top regions where *O. majorana* is mainly cultivated are Central Europe, Egypt, and Morocco. In the food industry, distilled oils and extracts of sweet marjoram are frequently applied as a spice and to increase storage stability and reduce microbial contamination. In the folk medicine, it is used for the treatment of respiratory or gastrointestinal disorders and urinary tract infection and also as a spasmolytic, antirheumatic, diuretic, and antiasthmatic remedies.^1,2^ Previous pharmacological investigations revealed the antioxidant, antiulcer, gastric secretory, antimicrobial, and antiplatelet activities of *O. majorana* extracts.^3,4^ In many studies, essential oil (EO) and its constituents, namely, terpinen-4-ol, *cis*-sabinene hydrate, linalool, sabinene, α-terpinene, α-terpineol, γ-terpinene, and *p*-cymene, have been shown to exert antibacterial and antifungal effects on a variety of bacterial and fungal strains, including some drug-resistant clinical isolates.^3,5−7^ It was also investigated how sweet marjoram and terpinen-4-ol affect the formation of single- and dual-species biofilms on food surfaces that could modify food quality and/or cause serious foodborne illnesses.^8^ With regard to the non-volatile compounds, diterpenes (carnosic acid and carnosol), cinnamic acid derivatives (rosmarinic acid, chlorogenic acid, etc.), and other phenolic acids, flavonoids, hydroquinones, as well as ursolic acid and oleanolic acid were isolated and identified in the hydroalcoholic and water extracts of *O. majorana*.^3,9^

The goal of the present study is to evaluate the chloroform extract of sweet marjoram, and compounds isolated from this extract for antimicrobial activity, ability to reverse bacterial multidrug resistance, and inhibit biofilm formation. The efflux mechanisms of bacteria are widely recognized as major components of resistance to many classes of antimicrobials. All bacterial plasma membranes contain efflux pumps (EPs), which are proteins that identify and extrude antibiotics into the environment before they reach their intended targets. One of the reasons why antimicrobial chemotherapy frequently fails is EP overexpression. The discovery of efflux pump inhibitors (EPIs) is a promising approach to improving the clinical performance of antibiotics.^10^ Bacterial biofilms are a microbial community consisting of bacterial cells in a matrix that are bonded to each other and to a surface. This community is embedded in a self-produced extracellular matrix. Bacteria that are embedded within biofilms are less sensitive to antibiotics. Among bacterial pathogens, *Staphylococcus aureus* and *Escherichia coli* are the most important; they frequently form biofilms and are able to colonize even the host tissue in the case of chronic infection.^11^

In our previous study, the antibacterial activities of EO of *O. majorana* and its constituents were investigated in detail.^12^ The present study was designed to examine the antimicrobial activity of non-volatile compounds because in the literature only a few references can be found regarding the antibacterial and antifungal effects of the compounds of *O. majorana* outside EO and its constituents. This study aimed to isolate the compounds of the chloroform extract, examine the effect of the extracts and compounds against bacteria and fungi and for their ability to reverse bacterial resistance and inhibit biofilm formation.

## Materials and Methods

### General Experimental Procedures

Melting points were determined using a Boetius apparatus. The optical rotations were measured using a JASCO P-2000 polarimeter (JASCO International, Co., Ltd., Hachioji, Tokyo, Japan). High-resolution mass spectrometry (HRMS) measurements were performed using a Thermo Velos Pro Orbitrap Elite (Thermo Fisher Scientific, Bremen, Germany) instrument via electron spray ionization (ESI) in the positive ion mode. The protonated molecular ion peaks were fragmented using the collision-induced dissociation (CID) method at a normalized collision energy of 35%. Data were obtained and processed using the MassLynx software. Helium was used as a collision gas in the CID experiments. Nuclear magnetic spectroscopy (NMR) spectra were recorded in CDCl_3_ or CD_3_OD on a Bruker Avance DRX 500 spectrometer (Bruker, Billerica, MA, USA) at 500 MHz (^1^H) and 125 MHz (^13^C). The signals of the deuterated solvents were taken as a references. Two-dimensional (2D) NMR measurements were performed using standard Bruker software. In the homonuclear correlation spectroscopy (^1^H–^1^H COSY), nuclear Overhauser effect spectroscopy (NOESY), heteronuclear single quantum coherence (HSQC), and heteronuclear multiple bond correlation (HMBC) experiments, gradient-enhanced versions were applied. Polyamide (MP Polyamide, 50–160 μM, MP Biomedicals, Irvine, CA, USA) was used for open-column chromatography (OCC) and rotational planar chromatography (RPC) was performed on silica gel 60 GF_254_ using a Chromatotron (Harrison Research). Flash chromatography (FC) was performed using a CombiFlash Rf+ Lumen via integrated UV, UV–vis, and ELS detection at a normal phase [silica 60, 0.045–0.063 mm (Molar Chemicals, Halásztelek, Hungary) and RediSep Rf Gold (Teledyne Isco, Lincoln NE, USA)] flash column. Sephadex LH-20 (25–100 μm, Sigma-Aldrich, St. Louis, Missouri, USA) was used for gel filtration (GF). LiChroprep RP-18 (15–25 μm, Merck) stationary phase was used for reversed-phase vacuum liquid chromatography (RP-VLC). Reversed-phase high-performance liquid chromatography (RP-HPLC) and normal-phase HPLC (NP-HPLC) separations were performed using a Shimadzu LC-10AS HPLC instrument equipped with a UV–vis detector (Shimadzu, Co., Ltd., Kyoto, Japan) over reversed-phase (RP-HPLC, LiChrospher RP-18, 5 μm, 250 × 4 mm) and normal-phase (NP-HPLC, LiChrospher Si60, 5 μm, 250 × 4 mm) columns, respectively. Preparative thin-layer chromatography (prep TLC) was performed using silica plates (20 × 20 cm silica gel 60 F_254_, Merck 105,554). TLC plates were visualized under a UV lamp at 254 nm and detected by spraying with concentrated sulfuric acid, followed by heating. Sigma-Aldrich Kft. and Molar Chemicals provided the chemicals used in this experiment.

### Plant Material

The dried shredded aerial parts of “Hungarian” variety *O. majorana* were purchased from a grower, Ferenc Okvátovity (Bátya, Hungary), who gathered the plant in July 2020. A voucher specimen no. 896 has been deposited in the Herbarium of the Institute of Pharmacognosy, University of Szeged, Szeged, Hungary.

### Isolation of the Compounds

The dried plant material (1.5 kg) was soaked in MeOH at room temperature overnight and then percolated with 17 L MeOH. The crude extract was evaporated to 1 L and then subjected to solvent–solvent partition with *n*-hexane (1 × 3 L) followed by CHCl_3_ (1 × 3 L). After concentration in vacuum, the residue of the CHCl_3_ phase was 15.76 g. This phase was chromatographed through a polyamide column (120 g) (OCC), with mixtures of H_2_O–MeOH (6:4, 4:6, 2:8, and 0:1) as eluents. Five fractions (Fr. I–V) were collected according to the eluents. Fraction II (3.75 g) was subjected to normal-phase flash chromatography (NP-FC) using a *n*-hexane–CHCl_3_ gradient system [linear from 0 to 50% CHCl_3_, time (*t*) = 45 min] and then eluted with MeOH (100%, *t* = 10 min). The collected fractions were combined based on TLC monitoring and seven subfractions (II/1–7) were obtained.

Fraction II/1 (84.5 mg) was further separated via RPC eluted with *n*-hexane–CHCl_3_ (1:1, 4:6, and 2:8), CHCl_3_–acetone (19:1, 9:1) and MeOH, affording seven subfractions (II/1a–g). Fractions II/1b and II/1c were purified by prep NP-TLC on 20 × 20 cm plates developed in *n*-hexane–CHCl_3_–MeOH (12:9:1). By this means, compound **1** (121 mg) was isolated. Fraction II/1c had another band on the prep TLC that was scratched, eluted with chloroform, and then further purified via NP-HPLC. HPLC separation was performed using *n*-hexane–EtOAc gradient system (linear from 0 to 75% EtOAc) as an eluent at a flow rate of 1 mL/min, affording compound **2** (0.4 mg). Fraction II/6 (105 mg) was subjected to NP-FC using a gradient system of *n*-hexane–EtOAc (linear from 25 to 50% EtOAc, *t* = 45 min) then eluted with MeOH (100%, *t* = 10 min). The collected fractions were combined based on TLC monitoring and six subfractions were obtained (II/6a–f). Fraction II/6a was fractionated via a next NP-FC using a *n*-hexane–EtOAc gradient system (linear from 0 to 30% EtOAc, *t* = 45 min), then eluted with 100% MeOH for 10 min, yielding subfractions II/6a_1–8_. Fraction II/6a_4_ showed two brown spots on the TLC plates; thus, it was purified via RP-HPLC using MeOH–H_2_O (4:1, isocratic, 1 mL/min) as an eluent, and compounds **3** (2 mg) and **4** (2.1 mg) were obtained in pure form. Fraction III was chromatographed on a silica gel column (90 g silica gel) with CHCl_3_–acetone (gradient 100:0, 97:3, 98:2, 85:15, 70:30, 60:40, and 50:50), then with 100% MeOH as eluents. A total of 14 fractions were gathered after TLC monitoring (III/1–14). Fractions III/6, 7, and 8 were subjected to NP-FC using cyclohexane–EtOAc–MeOH (95:5:0, 1:1:0, 0:1:1, and 0:0:1) separately. Fraction III/6–8 resulted in six subfractions (III/6–8/a–f); among them, subfraction III/6–8/d was subjected twice to GF on a Sephadex LH-20 with an elution of CH_2_Cl_2_–MeOH (1:1). The main fraction of this chromatography was purified via RPC with CHCl_3_–MeOH (1:0, 98:2, 96:4, 9:1, 8:2, 1:1, and 0:1) yielding compound **5** (18 mg), which crystallized from dimethyl sulfoxide (DMSO) and MeOH (1:1) as white crystals. Based on TLC monitoring, fraction III/6–8/f was purified via RP-VLC [AcNi–(H_2_O + 0.1% HCOOH) 25:75 up to 1:0], to yield compound **6** (4 mg) in the form of yellow crystals.

#### Majoranaquinone [3-(Hydroxymethyl)-2-methylnaphtho[2,3-*b*]furan-4,9-dione] (**1**)

Yellow crystals; mp 164–165 °C (lit. 166–168 °C);^19^ UV λ_max_ (log ε) 252 (3.867), 303 (3.818) and 403 (2.857) nm; ^1^H and ^13^C NMR (see Table 3); high-resolution electrospray ionization mass spectrometry (HRESIMS)-positive *m*/*z*: 243.0653 [M + H]^+^ (calcd for C_14_H_11_O_4_^+^, 243.0652), 225.0550 [M + H–H_2_O]^+^ (calcd for C_14_H_9_O_3_^+^, 225.0546).

#### 2,3-Dimethylnaphtho[2,3-*b*]furan-4,9-dione (**2**)

Yellow amorphous solid; ^1^H NMR (see Table 3); HRESIMS-positive *m*/*z*: 227.0701 [M + H]^+^ (calcd for C_14_H_11_O_3_, 227.0708).

#### 19-Hydroxyabieta-8,11,13-trien-7-one (**3**)

White amorphous solid; [α]_D_^25^ + 10.9 (*c* 0.1, MeOH); ^1^H and ^13^C NMR (see Table 4); HRESIMS positive *m*/*z*: 301.2163 [M + H]^+^ (calcd for C_20_H_29_O_2_, 301.2162).

#### 13,14-Seco-13-oxo-19-hydroxyabieta-8-en-14-al (**4**)

Colorless amorphous solid; [α]_D_^25^ + 77.5 (*c* 0.1, MeOH); ^1^H NMR and ^13^C (see Table 4); HRESIMS-positive *m*/*z*: 321.2428 [M + H]^+^ (calcd for C_20_H_33_O_3_, 321.2424).

#### 7-*O*-Methyleriodictyol (sterubin) (**5**)

White crystals, mp 214–6 °C (lit. 215 °C);^13^ [α]_D_^25^ – 3.0 (*c* 0.1, MeOH); ^1^H and ^13^C NMR data are identical with published data;^13^ HRESIMS-positive *m*/*z*: 303.0867 [M + H]^+^ (calcd for C_16_H_15_O_6_, 303.0863).

#### 5,6,4′-Trihydroxy-7,8,3′-trimethoxyflavone (majoranin) (**6**)

Yellow crystals; mp 224–225 °C (lit. 228–230 °C);^14^^1^H and ^13^C NMR data are in a good agreement with published data;^14^ HRESIMS-positive *m*/*z*: 361.0917 [M + H]^+^ (calcd for C_18_H_17_O_8_, 361.0918).

### Bacterial and Fungal Strains and Culture Conditions for Antimicrobial Assay

Gram-positive strains: *S. aureus* (ATCC 29213), *S. aureus* (MRSA) (ATCC 43300), *Staphylococcus epidermidis* (ATCC 12228), *Streptococcus agalactiae* (ATCC 13813), *Streptococcus pyogenes* (ATCC 19615), *Bacillus subtilis* (ATCC 6633), and *Enterococcus faecalis* (ATCC 29212) and standard Gram-negative strains *E. coli* (ATCC 35218), *E. coli* K-12 AG100 strain, *Klebsiella pneumoniae* (ATCC 700603), *Moraxella catarrhalis* (ATCC 25238), and *Pseudomonas aeruginosa* (ATCC 27853). The fungal strain *Candida albicans* (ATCC 10231), *Candida tropicalis* (ATCC 750), *Candida parapsilosis* (ATCC 22019), and *Nakaseomyces glabrata* (ATCC 2001) were used in this study. Bacterial cultures were grown on a standard Mueller–Hinton agar and fungal culture on RPMI plates (Diagnosticum Zrt.) at 37 °C under an aerobic environment overnight.

### Determination of Antibacterial Activity Using the Disc Diffusion Method

The disc diffusion method was employed to screen extracts and fractions for their antibacterial activity against standard bacterial and fungal strains to determine their inhibition zones. Concisely, the samples were dissolved in DMSO in 50 mg/mL concentration. The sterile filter paper discs [6 mm in diameter, Whatman antibiotic paper disc (Cytiva)] coated with 10 μL of the sample solutions were placed on top of the bacterial suspension (inoculums 0.5 McFarland, 1.5 × 10^8^ cfu mL^–1^). Discs containing antibiotic (ciprofloxacin) and antifungal (nystatin) were used as positive controls and DMSO as the negative control. Under aerobic conditions, the plates were incubated at 37 °C ±2 °C for 20 h. The diameters of inhibition zones caused by the compounds, including the disc, were measured and recorded in triplicate.^12^ For each of the three repetitions, an average zone of inhibition was calculated.

### Determination of Minimum Inhibitory Concentration Values

In accordance with the recommendations of the Clinical and Laboratory Standards Institute (CLSI), the minimum inhibitory concentration (MIC) of the samples was established using the microdilution method in a 96-well plate. The Mueller–Hinton broth was the medium used. Pure chemicals were evaluated at concentrations between 100 and 0.195 mM. Through a visual assessment, the MIC was established. The subinhibitory concentration of DMSO (1% v/v) was used as a solvent. The values are expressed as mean determined for three replicates from three independent experiments.^15^

### Bacterial Strains for Efflux Pump Inhibitory Assay

The wild-type *E. coli* K-12 AG100 [argE3 thi-1 rpsL xyl mtl Δ(gal-uvrB) supE44], expressing the AcrAB-TolC EP at its basal level and *E. coli* (ATCC 25922) strains were used as Gram-negative strains. As Gram-positive strains, the *S. aureus* (ATCC 25923) strain was investigated as a methicillin-susceptible reference and methicillin- and oxacillin-resistant *S. aureus* MRSA ATCC 43300 strains were used in the study.

### Real-Time Ethidium Bromide Accumulation Assay

Using a CLARIOstar Plus plate reader (BMG Labtech, UK) and the automated ethidium bromide (EB) method, the effect of compound **1** on EB accumulation was determined. The bacterial strain was first incubated until an optical density of 0.6 at 600 nm was achieved. Phosphate-buffered saline (PBS; pH 7.4) was used to wash the culture. After centrifuging the culture at 13,000*g* for 3 min, the cell pellet was re-suspended in PBS. Compound **1** was added to PBS containing a non-toxic concentration of EB (2 μg/mL) at concentrations of 500 and 1000 μM if the MIC was greater than 1000 μM (*E. coli* strains)^16^ and at MIC/2 concentration (*S. aureus* strains).^17^ A 96-well black microtiter plate (Greiner Bio-One Hungary Kft, Hungary) was then filled with 50 μL EB solution containing the test sample and 50 μL bacterial suspension (OD600 0.6). Carbonyl cyanide 3-chlorophenylhydrazone (CCCP) was used as a positive control at a concentration of 50 μM for both *E. coli* and *S. aureus* strains. The plates were then evaluated using a CLARIOstar plate reader and real-time fluorescence monitoring was performed every minute for 1 h at wavelengths of 525 and 615 nm for excitation and emission, respectively. Each experiment was conducted in triplicate. From the real-time data, the activity of the samples, namely, the relative fluorescence index (RFI) of the last time point (minute 60) of the EB accumulation assay, was calculated as follows
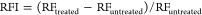
where RF_treated_ denotes the relative fluorescence (RF) at the last time point of EB retention curve in the presence of an inhibitor and RF_untreated_ denotes the RF at the last time point of the EB retention curve of the untreated control with the solvent control (DMSO).

### Inhibition of Biofilm Formation

*E. coli* strains (K-12 AG100 and ATCC 25922) and *S. aureus* strains (ATCC 25923 and MRSA 272123) were used as Gram-negative and Gram-positive bacteria. The dye crystal violet [CV; 0.1% (v/v)] was used to detect the development of biofilms.^18^ For *E. coli* or *S. aureus*, the initial inoculum was cultured in a Luria–Bertani broth (LB) (for *E. coli*) or in a Tryptic Soy broth (TSB) medium (for *S. aureus*) for an overnight period before being diluted to an OD600 of 0.1. Compound **1** was then added to 96-well microtiter plates together with the bacterial suspension at half the MIC or at 500 or 1000 μM. The final volume of each well was 200 μL and the positive controls were CCCP (*E. coli*) and thioridazine (TZ) (*S. aureus*). The plates were incubated at 30 °C for 48 h, with gentle stirring (100 rpm). After incubation, the TSB medium was discarded and the plates were washed with tap water to remove unattached cells. The wells were then filled with 200 μL CV and then incubated for 15 min at room temperature (24 °C). The following phase involved the removal of CV from the wells, washing of the plates with tap water and addition of 200 μL of 70% ethanol to the wells. A Multiskan EX ELISA plate reader (Thermo Labsystems, Cheshire, WA, USA) was used to measure OD600 to determine the biofilm formation. The biofilm formation inhibitory effect of the samples was expressed in the percentage (%) of biofilm formation decrease.

## Results and Discussion

### Bioassay-Guided Isolation of Compounds **1–6**

From the aerial parts of *O. majorana*, the MeOH extract was prepared, which was subjected to solvent–solvent partition, yielding *n*-hexane and chloroform extracts. The antimicrobial effect of the MeOH and *n*-hexane extracts, together with the EO obtained via hydrodistillation were previously reported.^12^ The present paper deals with the antimicrobial activity of the chloroform extract and its constituents. The chloroform extract exhibited the highest antibacterial and antifungal activities among the extracts and EO when tested by the disc diffusion method at a concentration of 50 mg/mL.^12^*S. aureus*, *S. aureus* MRSA, *S. epidermidis*, *C. albicans*, and *N. glabrata* proved to be the most susceptible strains for the chloroform extract (Table 1). To identify the compounds responsible for the activity, fractionation of the chloroform extract was checked via an antimicrobial assay.

**Table 1:** Antimicrobial Effect of the Chloroform Extract of *O. majorana* Determined Using the Disc Diffusion Method (Diameter, mm)^a^

	chloroform extract		ciprofloxacin		nystatin	
	mean	SD	mean	SD	mean	SD
Gram-positive						
*Staphylococcus aureus* ATCC 29213	20.0	0	30.0	0	NA	
*Staphylococcus aureus* MRSA ATCC 43300	22.0	0	27.3	0.58	NA	
*Staphylococcus epidermidis* ATCC 12228	20.0	0	34.0	0	NA	
*Streptococcus agalactiae* ATCC 13813	8.7	0.58	17.4	0.43	NA	
*Streptococcus pyogenes* ATCC 19615	10.0	1.0	18.2	0.81	NA	
*Enterococcus faecalis* ATCC 29212	0	0	20.1	1.13	NA	
*Bacillus subtilis* ATCC 6633	14.0	0	28.3	0.25	NA	
Gram-negative						
*Escherichia coli* ATCC 35218	0	0	30.2	1.04	NA	
*Escherichia coli* AG-100	0	0	30.0	0	NA	
*Klebsiella pneumoniae* ATCC 700603	4.7	4.04	24.4	1.30	NA	
*Pseudomonas aeruginosa* ATCC 27853	0	0	28.3	0.25	NA	
*Moraxella catarrhalis* ATCC 25238	16.3	1.53	30.0	0	NA	
Fungi						
*Candida albicans* ATCC 10231	17.0	0	NA		20.0	0
*Candida tropicalis* ATCC 750	6.0	5.29	NA		24.0	0
*Candida parapsilosis* ATCC 22019	9.7	0.58	NA		19.5	0.53
*Nakaseomyces glabrata* ATCC 2001	14.7	0.58	NA		20.8	0.12

a Samples were dissolved in DMSO at concentration 50 mg/mL; nystatin and ciprofloxacin was tested in 5 μg/disc; NA—not applicable.

The chloroform extract was separated via open column chromatography on polyamide to yield five fractions (frs. I–V); the antimicrobial activity of these fractions against the most susceptible strains, *S. aureus* MRSA, *M. catarrhalis*, and *C. tropicalis*, was investigated. The highest antibacterial and antifungal activities were demonstrated by fractions I and II (Table 2).

**Table 2:** Antimicrobial Effect of Fractions I–V of the Chloroform Extract Determined Using the Disc Diffusion Method (Diameter, mm)^a^

	Fr I		Fr II		Fr III		Fr IV		Fr V		CIP^b^		NY^c^	
microorganism	mean	SD	mean	SD	mean	SD	mean	SD	mean	SD	mean	SD	mean	SD
*S. aureus* MRSA ATCC 43300	27.7	0.58	33.3	0.58	11.7	0.58	9.0	0	13.0	0	27.3	0.58	NA	
*M. catarrhalis* ATCC 25238	9	0	14	0	0	0	0	0	14.0	0	30.0	0	NA	
*C. tropicalis* ATCC 750	12.0	0	23.0	0	0	0	0	0	12.0	0	NA		24.0	0

a Fr I–V were dissolved in DMSO at a concentration of 50 mg/mL.

b CIP—ciprofloxacin 5 μg/disc.

c NY—nystatin 5 μg/disc; NA—not applicable.

**Figure 1:**
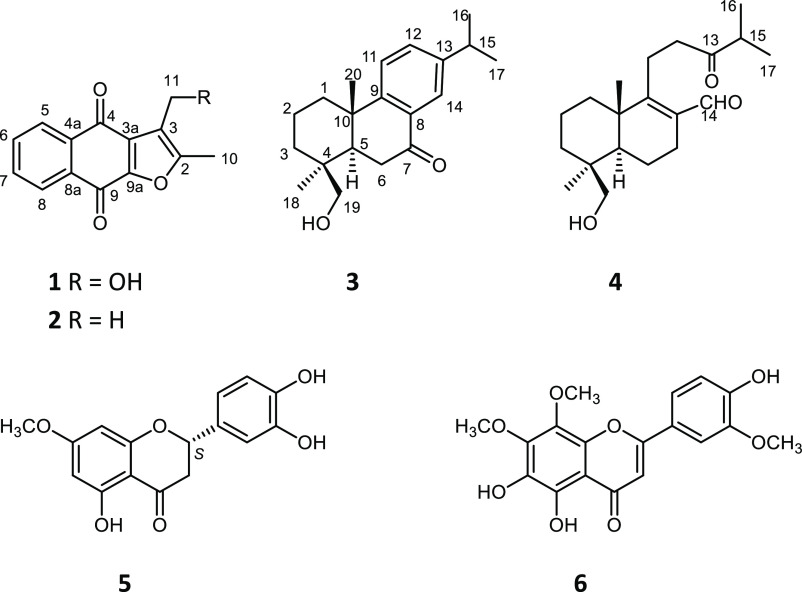
Structure of the isolated compounds **1**–**6**.

Further multistep chromatographic separation, including FC, GF, VLC, RPC, prep TLC, and HPLC, afforded six pure compounds (**1**–**6**) (Figure 1). The structure elucidation was performed via spectroscopic analysis, including 1D and 2D NMR (^1^H–^1^H COSY, HSQC, HMBC, and NOESY) and HRESIMS experiments.

### Structure Elucidation of Compounds **1–6**

Compound **1** was obtained as yellow crystals with no optical rotation [α]_D_^25^ 0 (*c* 0.1, MeOH). The UV spectrum of **1** demonstrated absorption maxima at 252, 303, and 403 nm. It gave the molecular formula C_14_H_10_O_4_, determined from the HRESIMS by the protonated molecular ion peak at *m*/*z* 243.0653 [M + H]^+^ (calcd for C_14_H_11_O_4_^+^, 243.0652). Analysis of the ^1^H NMR spectrum of **1** revealed the presence of a 1,2-disubstituted aromatic ring as signals were detected in the aromatic region of the spectrum at δ_H_ 8.12 dd (*J* = 7.5 and 1.5 Hz), 7.72 dt (*J* = 7.5 and 1.5 Hz), 7.75 dt (*J* = 7.5, 1.6 Hz), and 8.17 dd (*J* = 7.5 and 1.6 Hz). Furthermore, a downfield-shifted methyl signal at δ_H_ 2.40 s (3H) and a singlet signal at δ_H_ 4.60 with two proton intensities were detected.

The ^13^C-JMOD spectrum exhibited eight quaternary carbons, four methines, one methylene, and one methyl group in the molecule (Table 3). The ^1^H–^1^H COSY spectrum defined one structural fragment with correlated protons −CH=CH–CH=CH– (δ_H_ 8.12 dd, 7.72 dt, 7.75 dt, and 8.17 dd) (H-5–H-8). This four-carbon fragment, together with two keto groups (δ_C_ 183.2 and 172.9) and non-protonated sp^2^ carbons at δ_C_ 156.1 (C-2), 120.7 (C-3), 130.1 (C-3a), 132.8 (C-4a), 132.5 (C-8a), and 151.5 (C-9a), forms a disubstituted furano-1,4-naphtoquinone skeleton, as indicated by the HMBC correlations between H-5 (δ_H_ 8.12), C-4 (δ_C_ 183.2), and C-8a (δ_C_ 132.5), H-6 (δ_H_ 7.72) and C-4a (δ_C_ 132.8), H-7 (δ_H_ 7.75) and C-8a, and H-8 (δ_H_ 8.17) and C-9 (δ_C_ 172.9). One methyl group (δ_H_ 2.40 s, 3H) was placed at C-2 with regard to the HMBC cross-peaks of H_3_-10 with C-3 (δ_C_ 120.7) and C-2 (δ_C_ 156.1); contrarily, one hydroxymethyl group (δ_H_ 4.60 s, 2H) was placed at C-3 as indicated by the HMBC correlations between H_2_-11 and C-2, C-3, C-4 (δ_C_ 183.2) and C-3a (δ_C_ 130.1). Accordingly, the structure of compound **1** was elucidated as 3-(hydroxymethyl)-2-methylnaphtho[2,3-*b*]furan-4,9-dione and the trivial name majoranaquinone was given. This is the first report on isolation of this compound from the natural source; however, it was previously reported as a synthetic compound, prepared from bromonaphthoquinone in a two-step reaction. Its ability to inhibit indoleamine 2,3-dioxygenase-catalyzed oxidative degradation of l-tryptophan to *N*-formylkynurenine was tested, but no significant activity could be detected.^19^

**Table 3:** NMR Data of Compounds **1** and **2** [500 (^1^H) and 125 MHz (^13^C), CDCl_3_, δ ppm (*J* = Hz)]

	^1^H		^13^C
atom	1	2	1
**2**			156.1
**3**			120.7
**3a**			130.1
**4**			183.2
**4a**			132.8
**5**	8.12 dd (7.5, 1.5)	8.17 dd (7.5, 1.5)	127.0
**6**	7.72 dt (7.5, 1.5)	7.74 m	133.7
**7**	7.75 dt (7.5, 1.6)	7.74 m	134.3
**8**	8.17 dd (7.5, 1.6)	8.21 dd (7.5, 1.5)	127.0
**8a**			132.5
**9**			172.9
**9a**			151.5
**10**	2.40 s (3H)	2.45 s (3H)	12.2
**11**	4.60 s (2H)	2.34 s (3H)	55.0

Compound **2** was isolated as a yellow amorphous solid. Its HRESIMS spectrum exhibited a protonated molecular ion peak at *m*/*z* 227.0701 [M + H]^+^ (calcd for C_14_H_11_O_3_, 227.0708), indicating the molecular formula of C_14_H_10_O_3_. The ^1^H NMR data of compound **2** was very similar to those of majoranaquinone (**1**); the main difference was observed in the chemical shift and peak intensity of H-11, which was δ_H_ 2.34 s (3H) for **2** and δ_H_ 4.60 s (2H) for **1** (Table 3). These data indicated that the hydroxymethyl group was changed for a methyl group in **2** and its structure was accordingly elucidated as 2,3-dimethylnaphtho[2,3-*b*]furan-4,9-dione (Figure 1). This compound was previously synthesized by reacting lawsone with tiglic acid under Mitsunobu conditions. Compound **2** was moderately effective against the fungal strain *Magnaporthe grisea* (rice blast fungus).^20^ This is the first isolation of **2** from a natural source.

Furanonaphthoquinones are rarely occurring compounds in plants. Only a few such compounds were isolated from the species of the Asteraceae, Verbenaceae, Gesneriaceae, Bignoniaceae, Lamiaceae, and Acanthaceae families. Avicequinones, stenocarpoquinone, dehydro-iso-α-lapachone, α-ethylfurano-1,4-naphthoquinone, and maturone are the main representatives of this group of specialized metabolites.^21^ Among them, avicequinone B, isolated from mangrove plant (*Avicennia*), is promising for drug development owing to its anoikis-sensitizing activity in human lung cancer cells. Anoikis sensitization may help cancer therapies to prevent cancer metastasis.^22^

**Figure 2:**
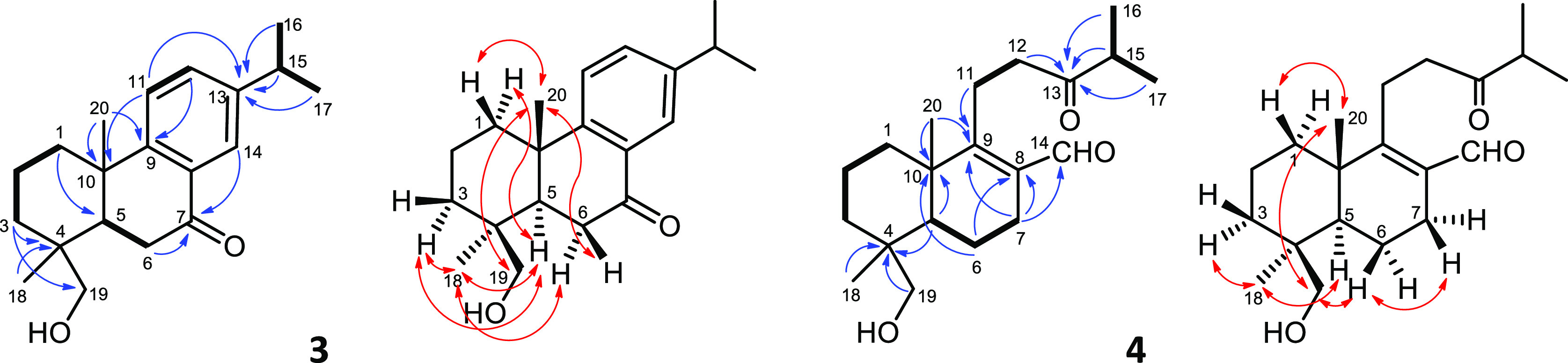
Key ^1^H–^1^H COSY (―), HMBC (blue →), and NOESY correlations (H red↔ H of compounds **3** and **4**.

Compound **3** was obtained as a white amorphous solid with an optical rotation value of [α]_D_^25^ + 10.9 (*c* 0.1, MeOH). Its molecular formula was determined to be C_20_H_28_O_2_ based on the HRESIMS ion peak at *m*/*z* 301.2163 [M + H]^+^ (calcd for C_20_H_29_O_2_, 301.2162). ^1^H NMR and ^13^C-JMOD spectra of **3** demonstrated characteristic signals of two tertiary methyls [δ_H_ 1.07 s (3H) and 1.27 s (3H); δ_C_ 26.5 and 24.1], an isopropyl [δ_H_ 1.27 d (*J* = 6.9 Hz) (6H) and 2.95 sept (*J* = 6.9 Hz); δ_C_ 23.9, 24.0, and 33.7], a hydroxymethyl [δ_H_ 3.67 and 3.92, both d (*J* = 10.8 Hz); δ_C_ 65.2], a keto group (δ_C_ 199.5) and a 1,3,4-trisubstituted aromatic ring [δ_H_ 7.33 d (*J* = 8.2 Hz), 7.42 dd (*J* = 8.2, 2.0 Hz), and 7.90 d (*J* = 2.0 Hz); δ_C_ 2 × 124.0, 125.1, 132.7, 147.0, and 153.7] (Table 4). Two sequences of correlated protons were extracted from the ^1^H–^1^H COSY spectrum: −CH_2_–CH_2_–CH_2_– (δ_H_ 2.41 brd, 1.62 dd, 1.78 dt, 1.74 m, 1.96 brd, and 1.11 dd) (A) and −CH–CH_2_– (δ_H_ 2.05 dd, 2.84 dd and 2.74 dd) (B). The connectivities of structural parts A and B, aromatic ring, keto, propyl, and methyl groups were established by evaluating the HMBC experiment. The ^2^*J*_C,H_ and ^3^*J*_C,H_ couplings of H_2_-6, H-14, and C-7; H_3_-18, H_2_-3 and C-4; H-11, H_3_-20, and C-10; H-15, H_3_-16, H_3_-17, H-11, and C-13; H-14, H-12, H_3_-20, and C-9; H_2_-19 and C-3; and H_2_-1 and C-5 demonstrated the planar structure 19-hydroxyabieta-8,11,13-trien-7-one (Figure 2). The relative stereochemistry of **3** was studied through the NOESY spectrum. The detected NOE-correlations of H_3_-20 with H-1β, H-6β, and H_3_-19 confirmed the β orientation of these protons and groups, whereas the Overhauser effects of H_3_-18 with H-5, H-6α, and H-3α proved α position of H-5 and 18-methyl group. Further NOESY correlations observed between H-5/H-1α, H-5/H-3α, and H-1α/H-2α allowed the differentiation of α or β protons at C-1–C-3 (Figure 2). The above findings were consistent with molecular formula 3, as presented in Figure 1. This compound was not described previously; only its stereoisomer, 18-hydroxyabieta-8,11,13-trien-7-one (in which the hydroxymethyl group is in the α-position), was reported from natural sources.^23^ The difference between compound **3** and 18-hydroxyabieta-8,11,13-trien-7-one was clearly demonstrated by the NMR data of methyls at δ_H_ 1.07 s and 0.93 s, δ_C_ 26.5 and 17.2 and hydroxymethyl groups at δ_H_ 3.92/3.67 and 3.45/3.15; and δ_C_.65.2 and 71.0, respectively.^23^

**Table 4:** NMR Data of Compounds **3** and **4** [500 MHz (^1^H), 125 MHz (^13^C), δ ppm (*J* = Hz), CDCl_3_]

	^1^H		^13^C	
position	**3**	**4**	**3**	**4**
1	*β* 2.41 br d (12.8)	*β* 1.93 m	38.2	35.6
	*α* 1.62 dd (12.8, 4.2)	*α* 1.23 dd (12.9, 4.6)		
2	*α* 1.78 dt (13.6, 3.0)	1.67 m	18.8	18.5
	*β* 1.74 m			
3	*α* 1.96 brd (13.7)	*α* 1.85 m	35.3	35.3
	*β* 1.11 dd (13.7, 4.5)	*β* 1.01 td (13.7, 4.1)		
4			38.4	39.0
5	2.05 dd (14.3, 3.8))	1.30 m	49.9	52.0
6	*α* 2.84 dd (18.1, 3.8)	*α* 1.90 m	36.3	18.2
	*β* 2.74 dd (18.1, 14.3)	*β* 1.40 dd (12.1, 5.9)		
7		*β* 2.43 dd (18.0, 5.9)	199.5	25.0
		*α* 2.09 ddd (18.0, 12.1, 7.2)		
8			124.0	132.4
9			153.7	166.9
10			38.0	41.1
11	7.33 d (8.2)	3.02 td (12.6, 4.5)	124.0	20.1
		2.52 m		
12	7.42 dd (8.2, 2.0)	2.74 ddd (17.7, 12.6, 4.5)	132.7	43.6
		2.58 m		
13			147.0	212.6
14	7.90 d (2.0)	10.03 s	125.1	193.0
15	2.95 sept (6.9)	2.56 m	33.7	41.0
16	1.27 d (6.9)	1.13 d (6.9)	23.9	18.4
17	1.27 d (6.9)	1.13 d (6.9)	24.0	18.4
18	1.07 s	1.05 s	26.5	26.9
19	3.92 d (10.8)	3.77 d (12.2)	65.2	65.5
	3.67 d (10.8)	3.53 d (12.2)		
20	1.27 s	1.09 s	24.1	20.6

Compound **4**, obtained as colorless amorphous solid, had an optical rotation data of [α]_D_^25^ + 77.5 (*c* 0.1, MeOH). Its HRESIMS spectrum suggested the molecular formula of C_20_H_32_O_3_ on the basis of the peak of the protonated molecule [M + H]^+^ displayed at *m*/*z* 321.2428 (calcd for C_20_H_33_O_3_, 321.2424). The ^13^C-JMOD spectrum of **4** indicated a diterpene core, which is built from four methyls (δ_C_ 18.4, 18.4, 20.6 and 26.9); eight methylenes (18.2, 18.5. 20.1, 25.0, 35.3, 35.6, 43.6, and 65.5); three methines (δ_C_ 41.0, 52.9, and 193.0), including an aldehyde (δ_C_ 193.0); and five quaternary carbons (δ_C_ 39.0, 41.1, 132.4, 166.9, and 212.6), including a keto group (δ_C_ 212.6) and tetrasubstituted olefinic bound (132.4, 166.9) (Table 4). The ^1^H–^1^H COSY spectrum of **4** revealed four sequences of correlated protons: −CH_2_–CH_2_–CH_2_– [δ_H_ 1.93 m, 1.23 dd, 1.67 (2H), 1.85 m, 1.01 td) (C-1–C-3], −CH–CH_2_–CH_2_– [δ_H_ 1.30, 1.90 m, 1.40 dd, 2.43 dd, 2.09 ddd] (C-5–C-7), −CH_2_–CH_2_– [δ_H_ 3.02 td, 2.52 m, 2.74 ddd, 2.58 m] (C-11–C-12), and −CH(CH_3_)_2_ (δ_H_ 2.56 m, 2 × 1.13 d) (C-15–C-17). The connectivities of COSY spin systems and quaternary carbons were determined by evaluating the HMBC spectrum. The heteronuclear ^2^*J*_C,H_ and ^3^*J*_C,H_ correlations of C-13 with H_2_-12 and H_3_-16/17; C-4 with H_3_-18, H_2_-19, and H-5; C-10 with H_2_-6, H-5, and H_3_-20; C-8 with H_2_-7 and H_2_-6; C-9 with H_2_-11, H_2_-7, and H_3_-20; and C-14 with H_2_-7 allowed the planar structure of compound **4**. The relative configuration of the stereogenic centers C-4, C-5, and C-10 of **4** was elucidated from the NOESY cross-peaks between H-5/H-18 and H_2_-19/H_3_-20; the same orientations as that of **3** were observed. NOE correlations also allowed the stereochemical assignment of protons of the methylene groups, indicated by the H_3_-20/H-1β, H-19/H-6β, H-6β/H-7β, and H-3α/H_3_-18 NOEs (Figure 2). The structure of compound **4** was, therefore, determined as 13,14-seco-13-oxo-19-hydroxyabieta-8-en-14-al (Figure 1). To the best of our knowledge, this is the first report on compound **4**; previously, only a few 13,14-seco-abietane derivatives were published from *Abies*(24) and *Chloranthus* species.^25,26^

Compound **5** was identified as 7-*O*-methyleriodictyol (sterubin) via analysis of its HRESIMS and 1D and 2D NMR spectra as well as comparison with the data published in the literature. This flavanone was previously isolated from *O. majorana*.^27^

Compound **6** was found to be identical in its ^1^H and ^13^C NMR characteristics and molecular composition with 5,6,4′-trihydroxy-7,8,3′-trimethoxyflavone (majoranin) isolated earlier from *Majorana hortensis*,^28^*Thymus vulgaris*,^29^*Origanum* × *intercedens*,^30^*Tanacetopsis mucronata*,^31^*Satureja atropatana*,^32^ and *Mentha* × *piperita citrata*.^33^

### Antibacterial and Antifungal Assay of Isolated Compounds **1–6**

The antimicrobial activity of the isolated compounds was evaluated against six Gram-positive and five Gram-negative bacterial strains. Majoranaquinone (**1**) had MIC values of 125 μM when tested against *S. aureus* ATCC 25923 and *S. aureus* MRSA ATCC 43300, 250 μM against *S. aureus* ATCC 29213 and *M. catarrhalis* ATCC 25238, and 1 mM against *E. faecalis* ATCC 29212. The highest activity was exhibited by **1** against *B. subtilis* ATCC 6633, with an MIC value of 7.8 μM (Table 5). Majoranaquinone (**1**) was found to be potent mainly against Gram-positive bacteria, except for *E. faecalis* ATCC 29212. Among Gram-negative strains only *M. catarrhalis* ATCC 25238 was sensitive (MIC, 250 μM). 19-Hydroxyabieta-8,11,13-trien-7-one (**3**), 13,14-seco-13-oxo-19-hydroxyabieta-8-en-14-al (**4**), sterubin (**5**), and majoranin (**6**) were assayed against the *S. aureus* ATCC 29213, *S. aureus* MRSA ATCC 43300, and *S. epidermidis* ATCC 12228 strains, but all of them were inactive (MIC > 1 mM). Compound **3** has a similar structure as the abietane diterpenes of sage and rosemary with an antimicrobial activity (carnosol, carnosic acid, rosmanol, epirosmanol, and isorosmanol), but they differ in the phenolic hydroxy groups and lactone ring, which are missing in **3**.

**Table 5:** MIC Values of the Isolated Compounds **1**, **3**–**6** against Gram-Positive and Gram-Negative Bacteria^a^^b^

	MIC (μM)					
bacterial strains	1	3	4	5	6	ciprofloxacin
Gram-positive						
*Staphylococcus aureus* ATCC 29213	**250**	>1000	>1000	>1000	>1000	0.3125
*Staphylococcus aureus* ATCC 25923	**125**	n.t.	n.t.	n.t.	n.t.	1.18
*Staphylococcus aureus* MRSA ATCC 43300	**125**	>1000	>1000	>1000	>1000	0.625
*Staphylococcus epidermidis* ATCC 12228	>1000	>1000	>1000	>1000	>1000	0.3125
*Enterococcus faecalis* ATCC 29212	1000	n.t.	n.t.	n.t.	n.t.	1.56
*Bacillus subtilis* ATCC 6633	**7.8**	n.t.	n.t.	n.t.	n.t.	0.0625
Gram-negative						
*Escherichia coli* ATCC 35218	>1000	n.t.	n.t.	n.t.	n.t.	0.019
*Escherichia coli* K-12 AG100	>1000	n.t.	n.t.	n.t.	n.t.	0.039
*Klebsiella pneumoniae* ATCC 700603	>1000	n.t.	n.t.	n.t.	n.t.	0.39
*Pseudomonas aeruginosa* ATCC 27853	>1000	n.t.	n.t.	n.t.	n.t.	0.195
*Moraxella catarrhalis* ATCC 25238	**250**	n.t.	n.t.	n.t.	n.t.	0.0625

a In bold, MIC values < 1000 μM.

b n.t. not tested.

Comparing the antimicrobial activity of majoranaquinone (**1**) with those of *O. majorana* EO constituents, it can be inferred that non-volatile compound **1** is several orders of magnitude more effective on some strains than the EO constituents. In our previous experiment under the same conditions, the most active volatile compounds, terpinene-4-ol and α-terpinene, exerted an antibacterial effect against *S. aureus* ATCC 29213 and *S. aureus* MRSA ATCC 43300 strains with MIC values of 60–61 mM,^15^ contrary to MIC values of 125 and 250 μM for **1**. Similar MIC values 0.25% (*v*/*v*) (=15 mM) were measured by other groups testing terpinene-4-ol against *S. aureus* strains ATCC-25923, ATCC-13150, NCTC 6571, and NCTC 29213 as well as clinical isolates.^34,35^

### Real-Time EB Accumulation Assay

The activity of majoranaquinone (**1**) on the EP function was evaluated via real-time fluorimetric assay on Gram-negative strains (*E. coli* ATCC 25922 and *E. coli* K-12 AG100) and Gram-positive strains (*S. aureus* ATCC25923 and *S. aureus* MRSA 43300), applying EB. Because EB is a substrate of the bacterial AcrB EP, the intracellular accumulation of EB provides information on the inhibition of the AcrAB-TolC system. Majoranaquinone (**1**) was evaluated at concentrations of 500 and 1000 μM against *E. coli* strains, as it demonstrated no antibacterial activity, whereas the MIC/2 concentration (62.5 μM) was applied when tested on *S. aureus* strains. Carbonyl cyanide 3-chlorophenylhydrazone (CCCP) and reserpine (RES) were used as positive controls at sub-MIC concentrations of 50 and 25 μM, respectively. The RFI was determined based on the means of RF units (Table 6). In the real-time EB accumulation assay, RFI values higher than the untreated control indicated an efflux pump inhibitory (EPI) effect. Majoranaquinone (**1**) was found to be effective on model Gram-negative bacterial strains; its RFI values of 2.91 (500 μM) and 2.52 (1000 μM) were higher than those of the positive control CCCP at 50 μM (RFI = 1.99) on *E. coli* ATCC 25922 strains. Testing on Gram-positive bacterial strains, majoranaquinone (**1**) exerted an efflux pump inhibition only on the *S. aureus* MRSA strain at an RFI value of 0.10 (positive control, 0.13). Efflux pump inhibitory activity could not be detected on *E. coli* K-12 AG100 and *S. aureus* ATCC 25923 strains (Table 6).

**Table 6:** RFI of Majoranaquinone (**1**) against *E. coli* and *S. aureus* Strains

	concentration	mean	SD	RFI
*E. coli* ATCC 25922				
compound **1**	500 μM	143,760	19166	**2.91**
compound **1**	1000 μM	129395	13.835	**2.52**
CCCP^a^	50 μM	109,847	2718	**1.99**
*E. coli* K-12 AG100				
compound **1**	500 μM	43,607	1810	–0.08
compound **1**	1000 μM	39,996	1248	–0.15
CCCP	50 μM	67,565	2360	**0.43**
*S. aureus* ATCC 25923				
compound **1**	62.5 μM	31,874	817	–0.28
RES^b^	25 μM	54,552	2682	**0.23**
*S. aureus* MRSA 43300				
compound **1**	62.5 μM	46,477	4048	**0.10**
RES	25 μM	47,555	3218	**0.13**

a CCCP carbonyl cyanide 3-chlorophenylhydrazone.

b RES reserpine.

The main constituents of the marjoram EO were also tested via the same assay on *E. coli* and *S. aureus* strains;^12^ therefore, the efflux pump inhibitory activities of volatile compounds and majoranaquinone (**1**) are comparable. On the *E. coli* ATCC 25922 strain, only sabinene exhibited a weak activity (RFI = 0.25), whereas on *S. aureus* MRSA ATCC 43300, only sabinene hydrate (RFI = 0.27) was effective at a concentration of 100 μM. Similar to majoranaquinone (**1**), the volatile compounds were ineffective as inhibitors of *E. coli* K-12 AG100 and *S. aureus* ATCC 25923 EPs.

### Biofilm Formation Inhibitory Effect of Compound **1**

**Figure 3:**
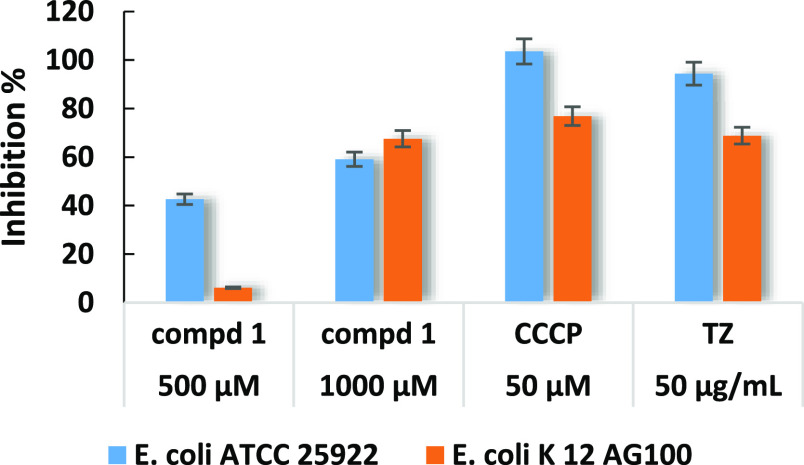
Biofilm formation inhibitory activity of majoranaquinone (**1**) on*E. coli* ATCC 25922 and*E. coli* K-12 AG100 strains.

**Figure 4:**
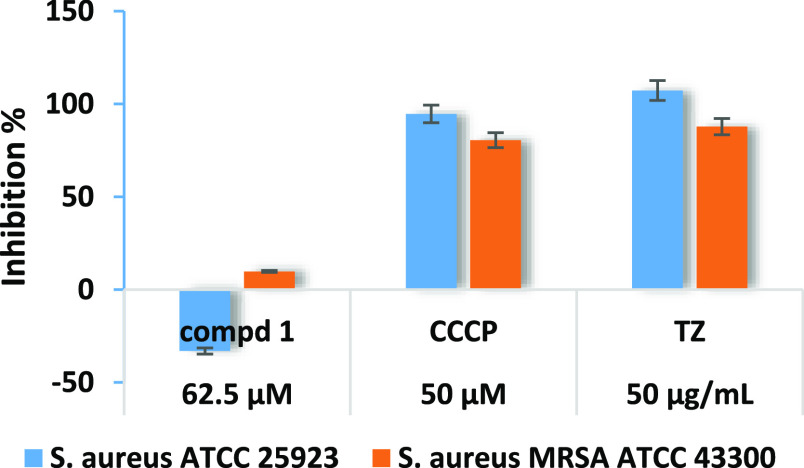
Biofilm formation inhibitory activity of majoranaquinone (**1**) on *S. aureus* ATCC 25923 and *S. aureus* MRSA ATCC 43300 strains; the concentration of compound **1** was MIC/2.

The inhibitory effect of majoranaquinone (**1**) treatment on biofilm formation was evaluated using the crystal violet method on *E. coli* and *S. aureus* bacteria. On *E. coli* strains, concentrations of 500 and 1000 μM were used in the anti-biofilm assay, whereas on *S. aureus* strains, a concentration of 62.5 μM was used. The positive controls were CCCP and TZ. The inhibition of biofilm formation was expressed in percentage; in general, values over 30% were considered to indicate significantly high inhibitory effects.^36^ As presented in Figures 3 and 4, majoranaquinone (**1**) significantly inhibited the biofilm formation of both *E. coli* strains, even at 500 μM; the inhibition percentages of *E. coli* ATCC25922 and *E. coli* K-12 AG100 were measured to be 42.62 and 6.14%, respectively. This activity was more pronounced at a higher concentration; at 1000 μM, **1** exhibited 59.10% inhibition against *E. coli* ATCC25922 and 67.56% against *E. coli* K-12 AG100 (Figure 3). In the case of *S. aureus* strains, no anti-biofilm activities could be detected (Figure 4).

Comparing the biofilm inhibitory activity of majoranaquinone (**1**) with those of the EO constituents of *O. majorana*,^12^ it can be observed that terpinene-4-ol, γ-terpineol, sabinene, sabinene hydrate, and linalool exert the same effects (inhibition, 37.80–55.97%) as that of **1** on *E. coli* ATCC 25922 strain; however, the volatile compounds were inactive on the resistant *E. coli* K-12 AG100 strain. Contrary to majoranaquinone (**1**), the formation of *S. aureus* MRSA ATCC 43300 biofilm was inhibited by terpinene-4-ol, sabinene, sabinene hydrate, and linalool (inhibition, 28.87–86.26%), but on the sensitive *S. aureus* ATCC 25923, they were ineffective.^12^

Several research groups have investigated the role of EPs in bacterial biofilm formation. EPs play different roles in biofilm formation; therefore, inhibition of their function could also inhibit biofilm formation. Many studies have demonstrated that some EPIs significantly reduce the development of biofilm in certain bacterial species.^37^ The same was also observed in our study; majoranaquinone (**1**) inhibited both EP and formation the biofilms of *E. coli* ATCC 25922.

## Conclusions

As a result of our bioactivity-guided isolation furanonaphthoquinones [majoranaquinone (**1**) and 2,3-dimethylnaphtho[2,3-*b*]furan-4,9-dione (**2**)], diterpenes [19-hydroxyabieta-8,11,13-trien-7-one (**3**) and 13,14-seco-13-oxo-19-hydroxyabieta-8-en-14-al (**4**)] and flavonoids [sterubin (**5**) and majoranin (**6**)] were isolated from the chloroform extract of the aerial parts of *O. majorana*. The chloroform phase exhibited pronounced antibacterial activity, especially against *S. aureus*, *S. aureus* MRSA, *S. epidermidis*, *C. albicans*, and *N. glabrata*, and among the isolated compounds, majoranaquinone (**1**) was found to be responsible for these activities as it demonstrated significant antibacterial activities against *B. subtilis*, *M. catarrhalis*, and different *Staphylococcus* strains (MIC, 7.8 μM–1 mM). The measured activities of **1** exceeded the antibacterial effects of the EO components, terpinene-4-ol, linalool, sabinene, sabinene hydrate, α-terpinene, and γ-terpinene.^15^ In the EPI assay, majoranaquinone (**1**) demonstrated remarkable activities on *E. coli* ATCC 25922 and *S. aureus* MRSA strains with RFI values close to that of the positive control. In this respect, compound **1** was more effective than any of the EO components on *E. coli* ATCC 25922 bacteria.^15^ The biofilm inhibitory activity of majoranaquinone (**1**) was also confirmed on *E. coli* and *S. aureus* strains. Against the biofilm formation of *E. coli* ATCC 25922, almost the same activities were observed among volatile compounds (γ-terpinene, terpinene-4-ol, sabinene, sabinene hydrate, and linalool) and **1**. Contrarily, against the biofilm formation of *E. coli* K-12 AG100, only majoranaquinone (**1**) was effective. *S. aureus* MRSA biofilm formation was more efficiently inhibited by the EO constituents than by **1**.

In summary, our findings indicate that besides EO, the non-volatile compounds, especially majoranaquinone (**1**), should also be considered in the assessment of the antimicrobial effect of *O. majorana*. The antimicrobial potency of **1** is comparable to that of plant products that are held to be the most effective, such as phenolic acids, flavonoids, and quinones.^38^ The MIC values of majoranaquinone (**1**) is in the same concentration range than that of caffeic acid and rosmarinic acid, but its effect is higher than that of e.g. quercetin and catechin.^39−41^ Furthermore, the triple effect (antimicrobial, efflux pump, and biofilm formation inhibitory) of **1** can also be highlighted. It would be worthwhile in the future to achieve studies to reveal the mechanism of action of the antimicrobial effect of majoranaquinone (**1**) and to map its synergism with volatile components and standard antibiotics.

## Supporting Information Available

^1^H NMR, ^13^C NMR JMOD, two-dimensional NMR, and HRESIMS spectra of compounds **1**–**6**, source data for determination of MIC values, RFI, and biofilm inhibitory activity of majoranaquinone (**1**) (PDF)
