# Supplementary material for: Furanonaphthoquinones, Diterpenes, and Flavonoids from Sweet Marjoram and Investigation of Antimicrobial, Bacterial Efflux, and Biofilm Formation Inhibitory Activities
Source: ACS Omega. 2023 Sep 14;8(38):34816–25. doi: 10.1021/acsomega.3c03982 (PMC10536869; doi:10.1021/acsomega.3c03982)
Supplement: Supplementary file 1 — ao3c03982_si_001.pdf [file ao3c03982_si_001.pdf]

Supporting information

Furanonaphthoquinones, diterpenes and flavonoids from sweet marjoram and investigation of antimicrobial, bacterial efflux and biofilm formation inhibitory activities

Tasneem Sultan Abu Ghazal,[1] Katalin Veres,[1] Lívia Vidács,[1] Nikoletta Szemerédi,[2] Gabriella Spengler,[2] Róbert Berkecz,[3] Judit Hohmann[1,4,5,*]

[1] Institute of Pharmacognosy, University of Szeged, H-6720 Szeged, Hungary

[2] Department of Medical Microbiology, Albert Szent-Györgyi Health Center and Albert Szent-Györgyi Medical School, University of Szeged, H-6725 Szeged, Hungary

[3] Institute of Pharmaceutical Analysis, University of Szeged, 6720 Szeged, Hungary

[4] Interdisciplinary Centre for Natural Products, University of Szeged, H-6720 Szeged, Hungary

[5] ELKH-USZ Biologically Active Natural Products Research Group, University of Szeged, H-6720 Szeged, Hungary

CONTENT

Figure 1. ^{1}H NMR spectrum of compound **1** ($CDCl_3$, 500 MHz) 2

Figure 2. ^{13}C NMR JMOD spectrum of compound **1** ($CDCl_3$, 125 MHz) 2

Figure 3. ^{1}H-^{1}H COSY spectrum of compound **1** ($CDCl_3$, 500 MHz) 3

Figure 4. HSQC spectrum of compound **1** ($CDCl_3$, 125/500 MHz) 3

Figure 5. HMBC spectrum of compound **1** ($CDCl_3$, 125/500 MHz)........ 4

Figure 6. ^{1}H NMR spectrum of compound **2** ($CDCl_3$, 500 MHz)........ 4

Figure 7. ^{1}H NMR spectrum of compound **3** ($CDCl_3$, 500 MHz)........ 5

Figure 8. ^{13}C NMR JMOD spectrum of compound **3** ($CDCl_3$, 125 MHz)........ 5

Figure 9. ^{1}H-^{1}H COSY spectrum of compound **3** ($CDCl_3$, 500 MHz) 6

Figure 10. HSQC spectrum of compound **3** ($CDCl_3$, 125/500 MHz)........ 6

Figure 11. HMBC spectrum of compound **3** ($CDCl_3$, 125/500 MHz)........ 7

Figure 12. NOESY spectrum of compound **3** ($CDCl_3$, 500 MHz) 7

Figure 13. ^{1}H NMR spectrum of compound **4** ($CDCl_3$, 500 MHz)........ 8

Figure 14. ^{13}C NMR JMOD spectrum of compound **4** ($CDCl_3$, 125 MHz)........ 8

Figure 15. ^{1}H-^{1}H COSY spectrum of compound **4** ($CDCl_3$, 500 MHz) 9

Figure 16. HSQC spectrum of compound **4** ($CDCl_3$, 125/500 MHz)........ 9

Figure 17. HMBC spectrum of compound **4** ($CDCl_3$, 125/500 MHz)........ 10

Figure 18. NOESY spectrum of compound **4** ($CDCl_3$, 500 MHz) 10

Figure 19. ^{1}H NMR spectrum of compound **5** (DMSO-d_6, 500 MHz)........ 11

Figure 20. ^{13}C NMR JMOD spectrum of compound **5** (DMSO-d_6, 125 MHz 11

Figure 21. ^{1}H NMR spectrum of compound **6** (DMSO-d_6, 500 MHz)........ 12

Figure 22. ^{13}C NMR JMOD spectrum of compound **6** (DMSO-d_6, 125 MHz)........ 12

Figure 23. HRESIMS of compound **1**........ 13

Figure 24. HRESIMS of compound **2**........ 13

Figure 25. HRESIMS of compound **3**........ 14

Figure 26. HRESIMS of compound **4**........ 14

Figure 27. HRESIMS of compound **5**........ 15

Figure 28. HRESIMS of compound **6**........ 15

Table 1. Minimum inhibitory concentration (MIC) values of majoranaquinone (**1**) 16

Table 2. RFI of majoranaquinone (**1**) against *E. coli* and *S. aureus* strains........ 16

Table 3. Biofilm formation inhibitory activity of majoranaquinone (**1**) on *E. coli* 16

Table 4. Biofilm formation inhibitory activity of majoranaquinone (**1**) on *S. aureus* 17

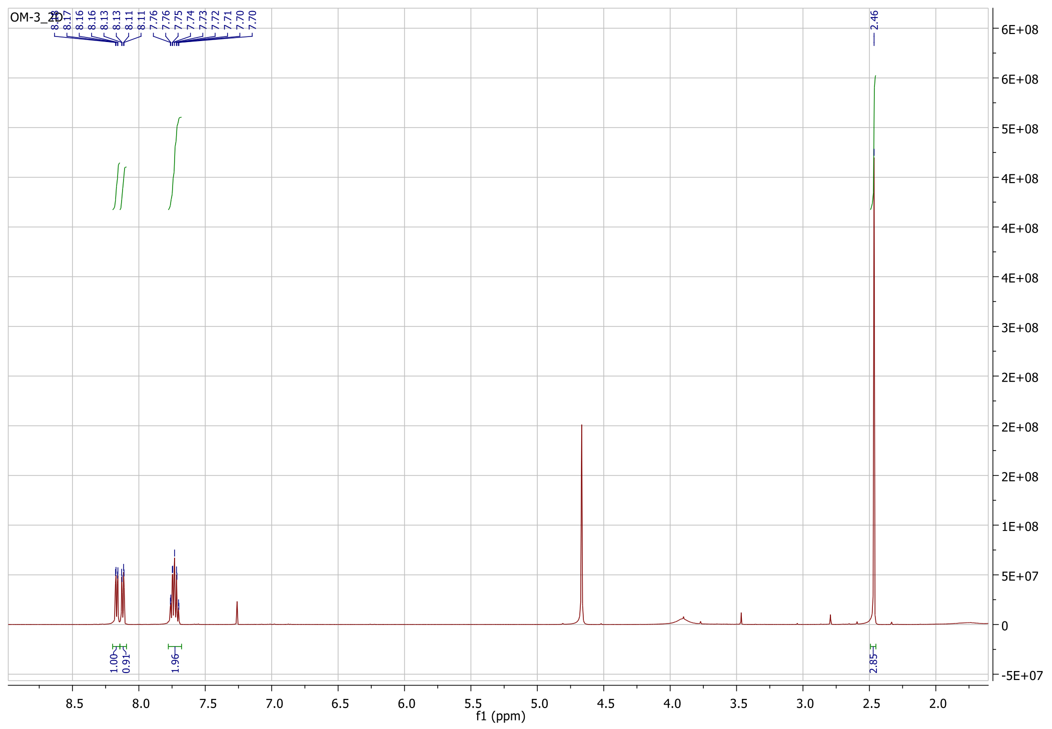

Figure 1. ^{1}H NMR spectrum of compound **1** ($CDCl_3$, 500 MHz)

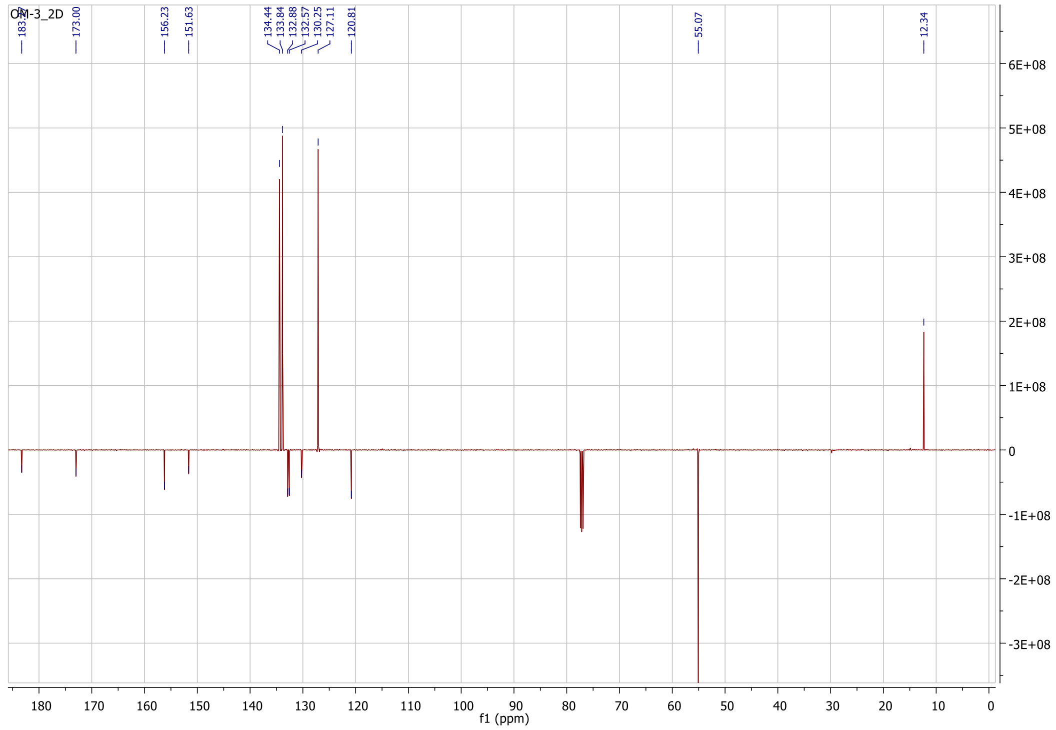

Figure 2. ^{13}C NMR JMOD spectrum of compound **1** ($CDCl_3$, 125 MHz)

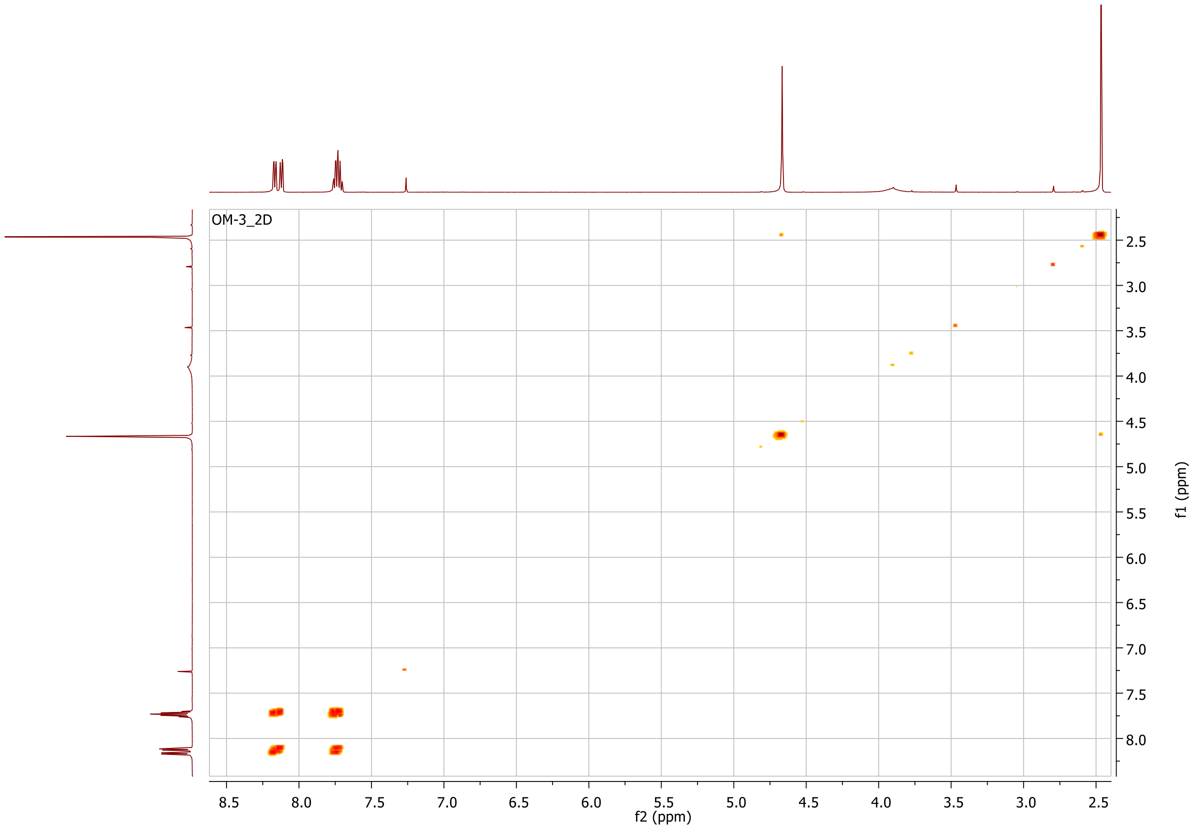

Figure 3. ^{1}H-^{1}H COSY spectrum of compound **1** ($CDCl_3$, 500 MHz)

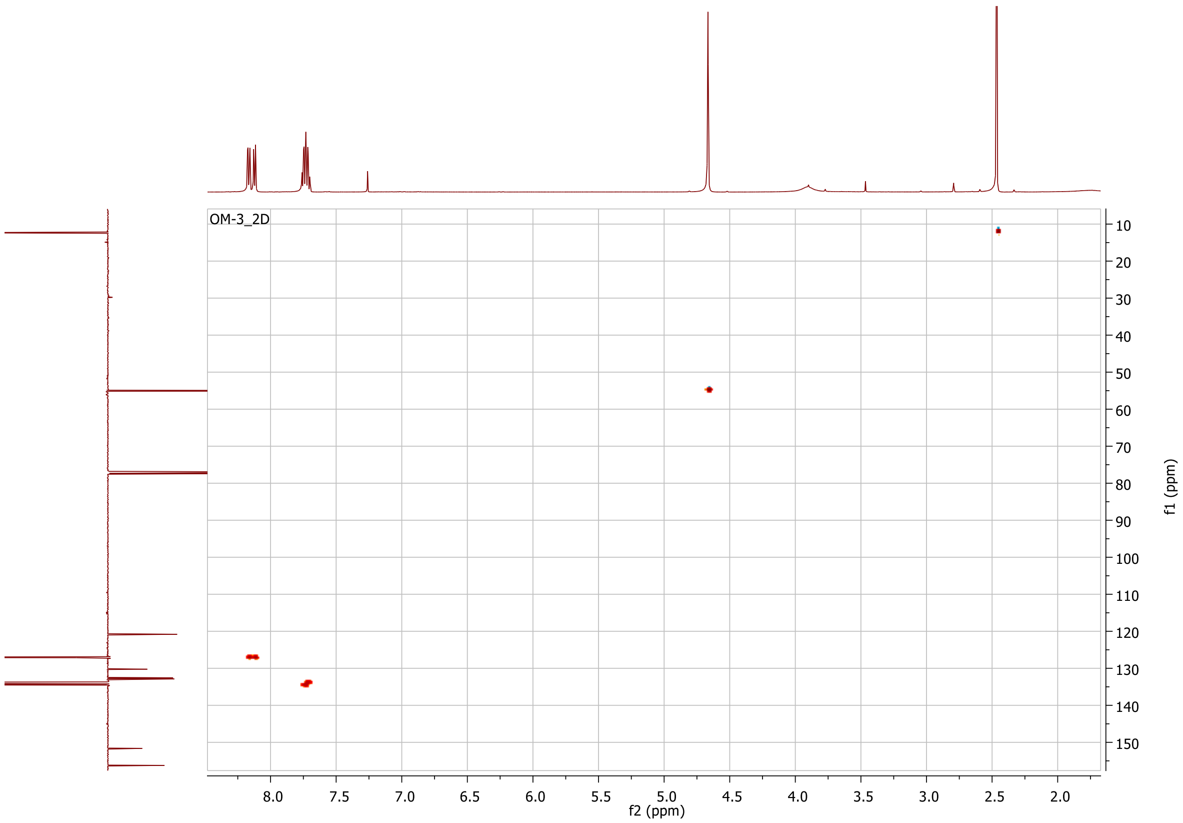

Figure 4. HSQC spectrum of compound **1** ($CDCl_3$, 125/500 MHz)

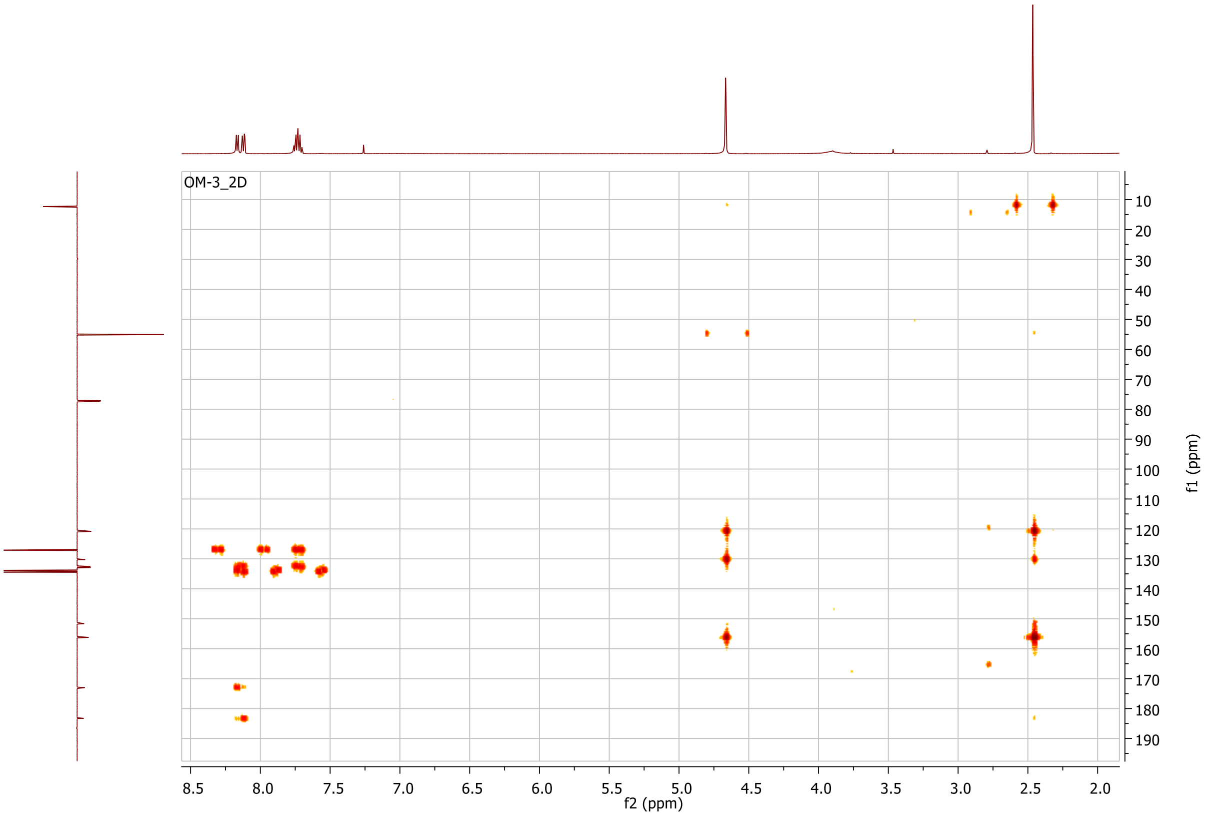

Figure 5. HMBC spectrum of compound **1** ($CDCl_3$, 125/500 MHz)

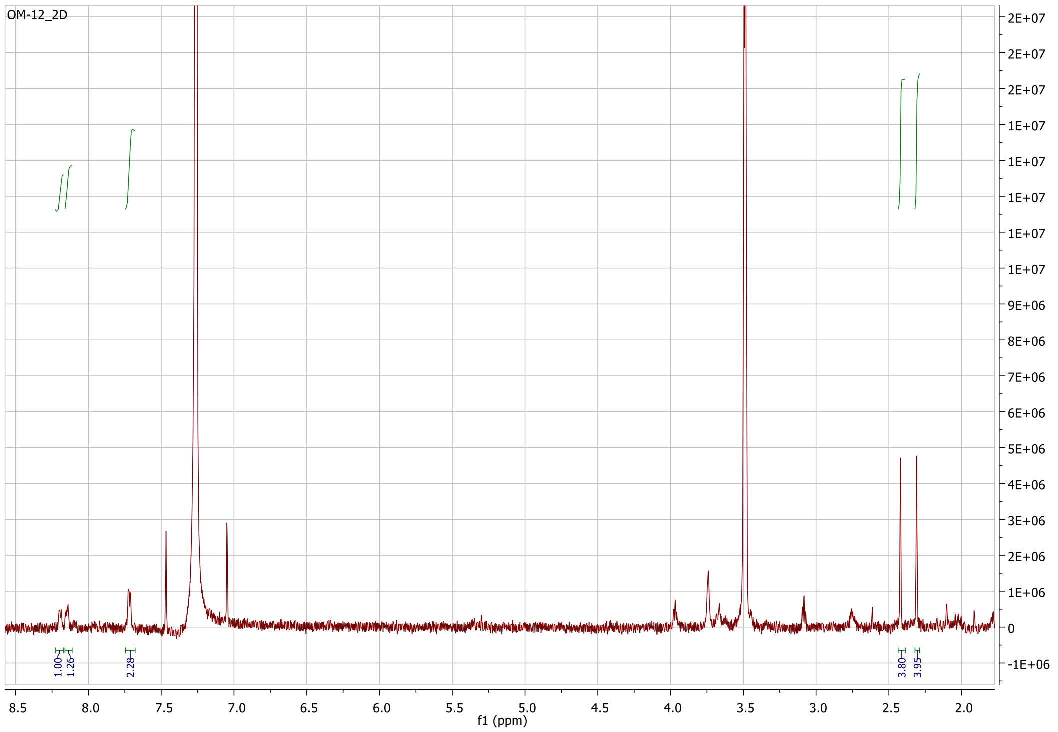

Figure 6. ^{1}H NMR spectrum of compound **2** ($CDCl_3$, 500 MHz)

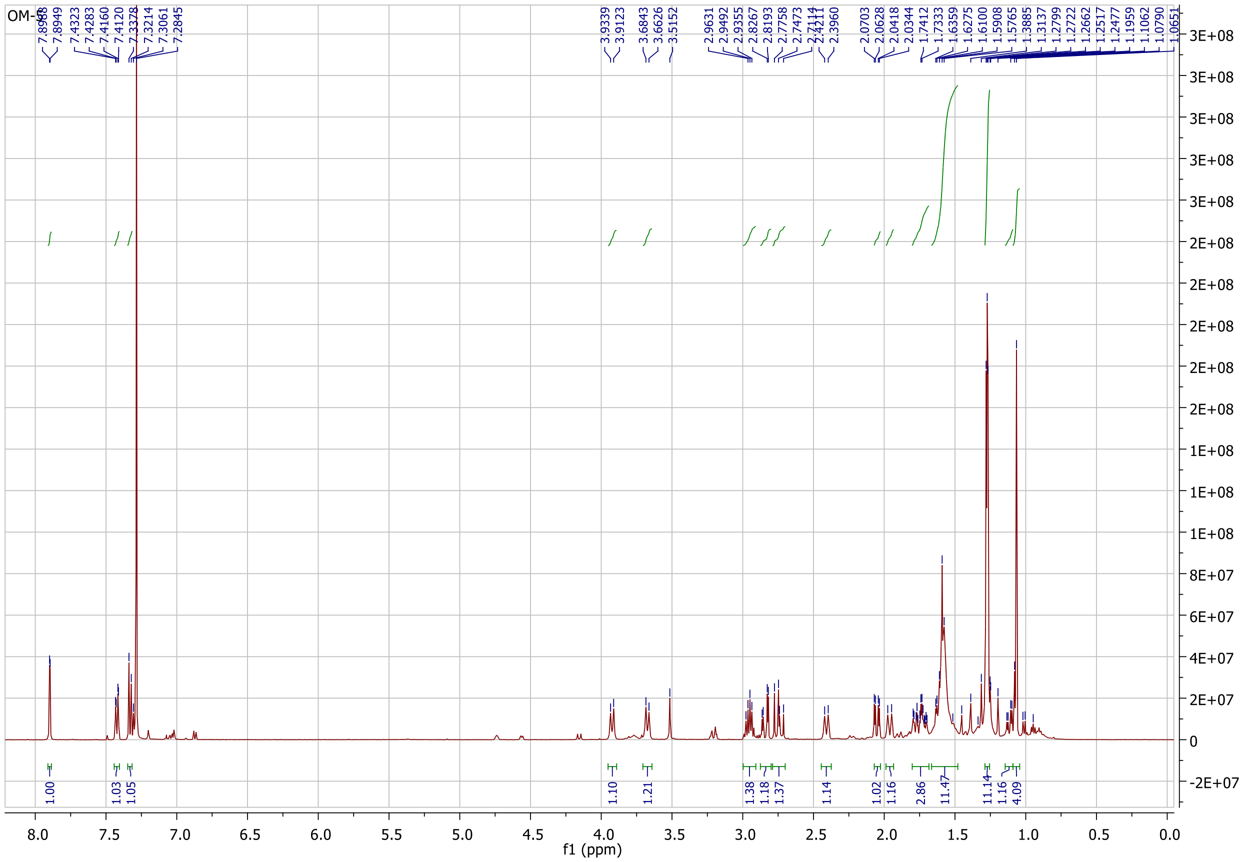

Figure 7. ^{1}H NMR spectrum of compound **3** ($CDCl_3$, 500 MHz)

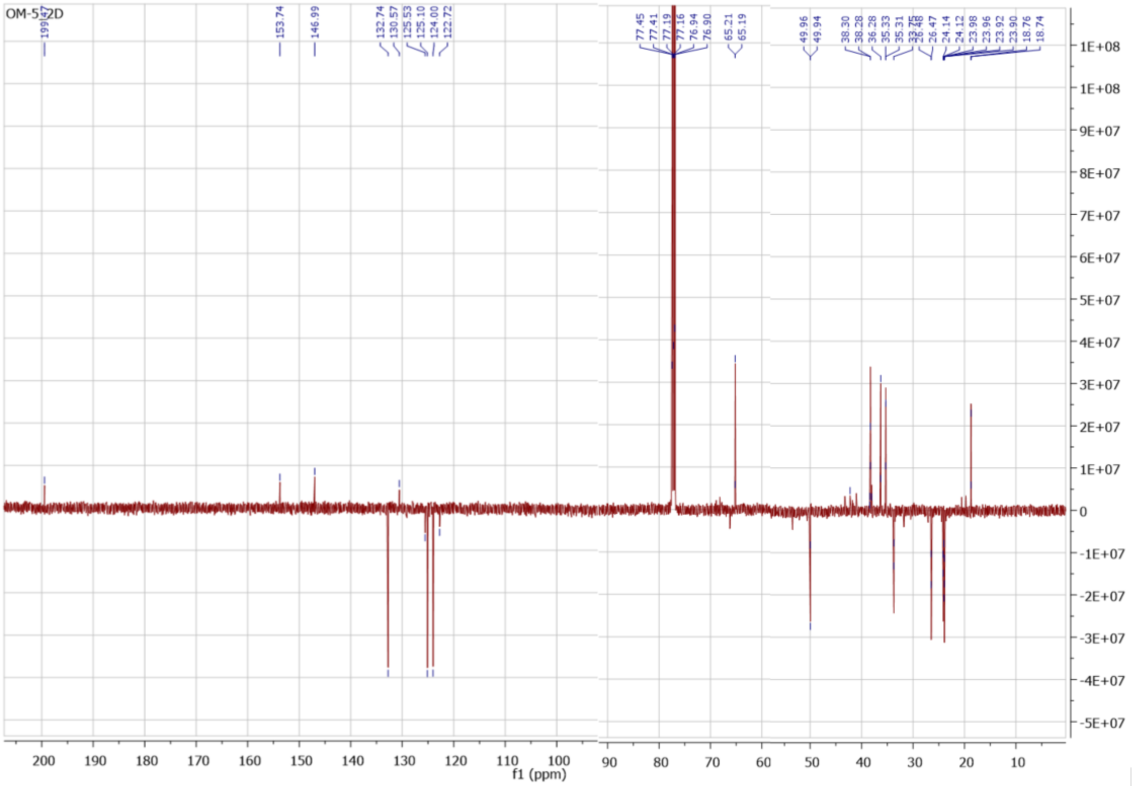

Figure 8. ^{13}C NMR JMOD spectrum of compound **3** ($CDCl_3$, 125 MHz)

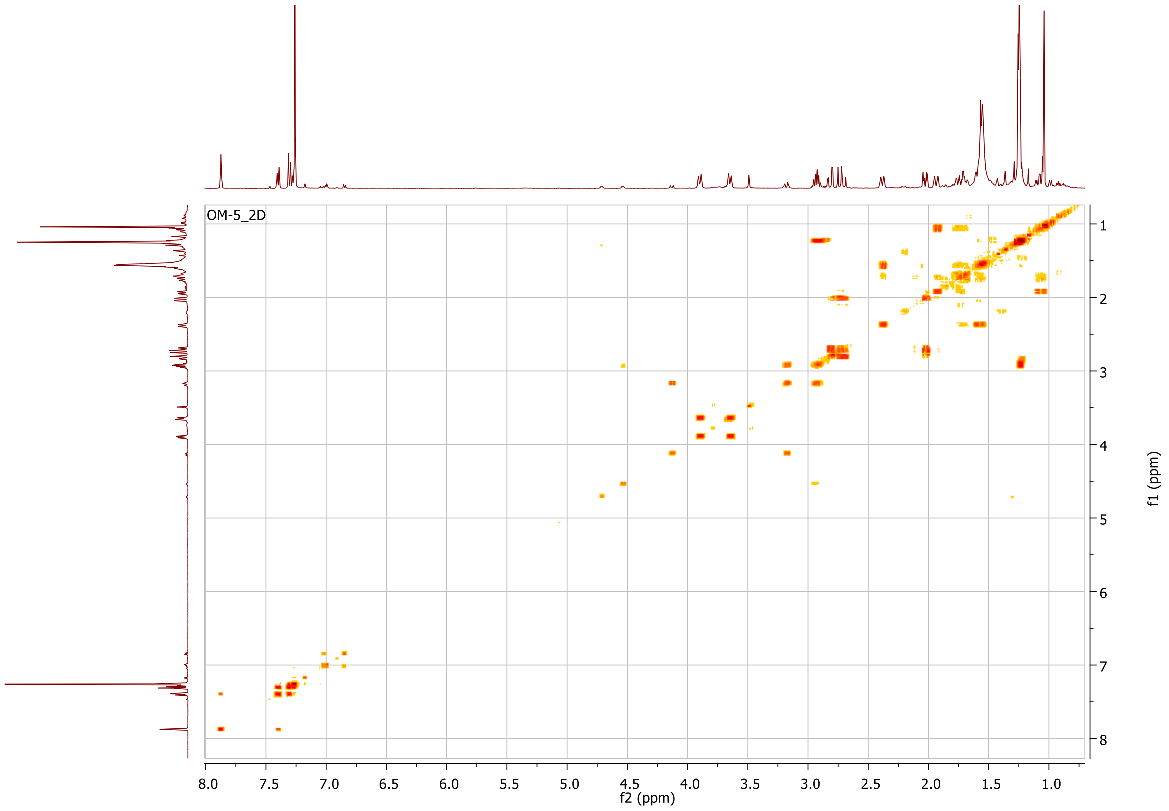

Figure 9. ^{1}H-^{1}H COSY spectrum of compound **3** ($CDCl_3$, 500 MHz)

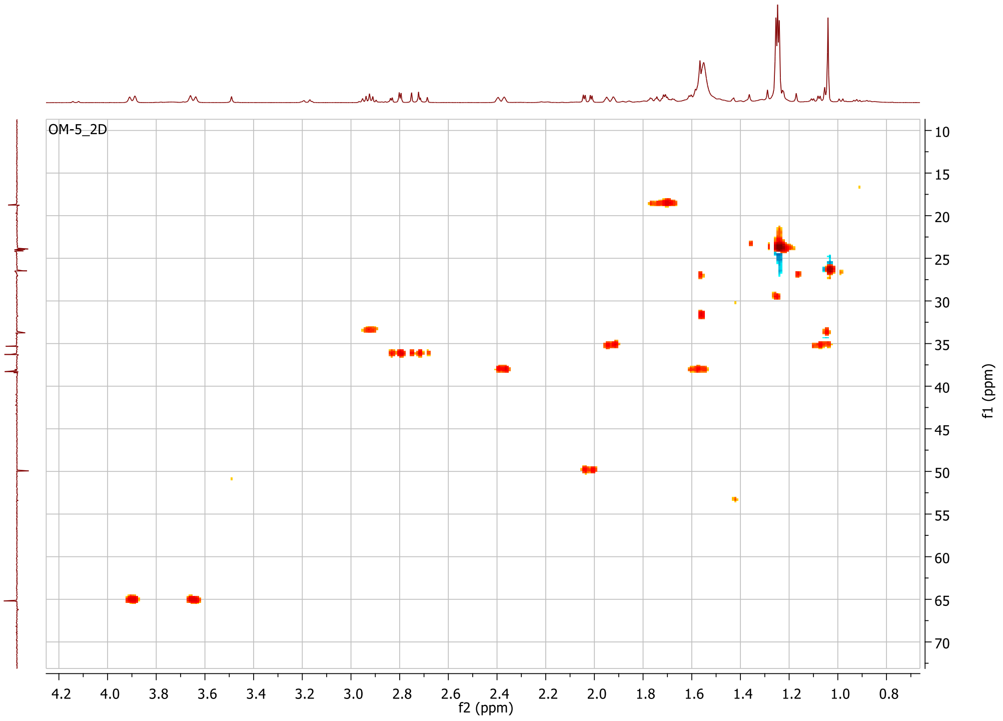

Figure 10. HSQC spectrum of compound **3** ($CDCl_3$, 125/500 MHz)

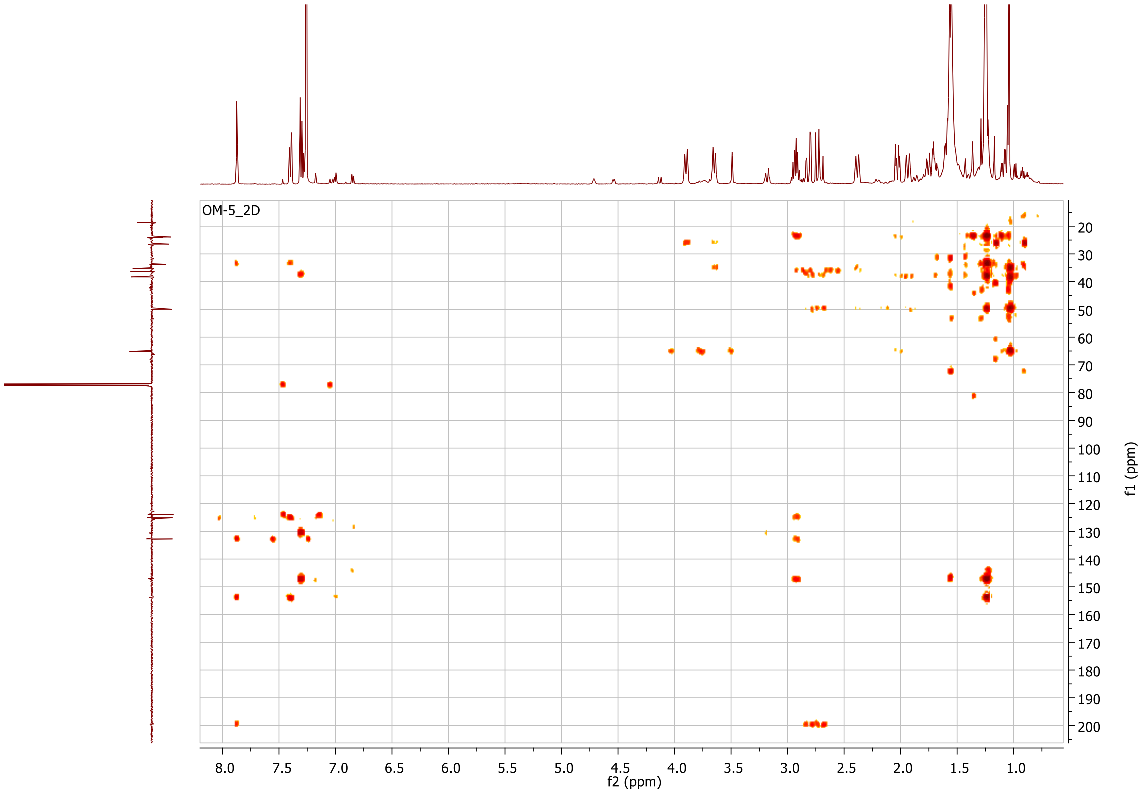

Figure 11. HMBC spectrum of compound **3** ($CDCl_3$, 125/500 MHz)

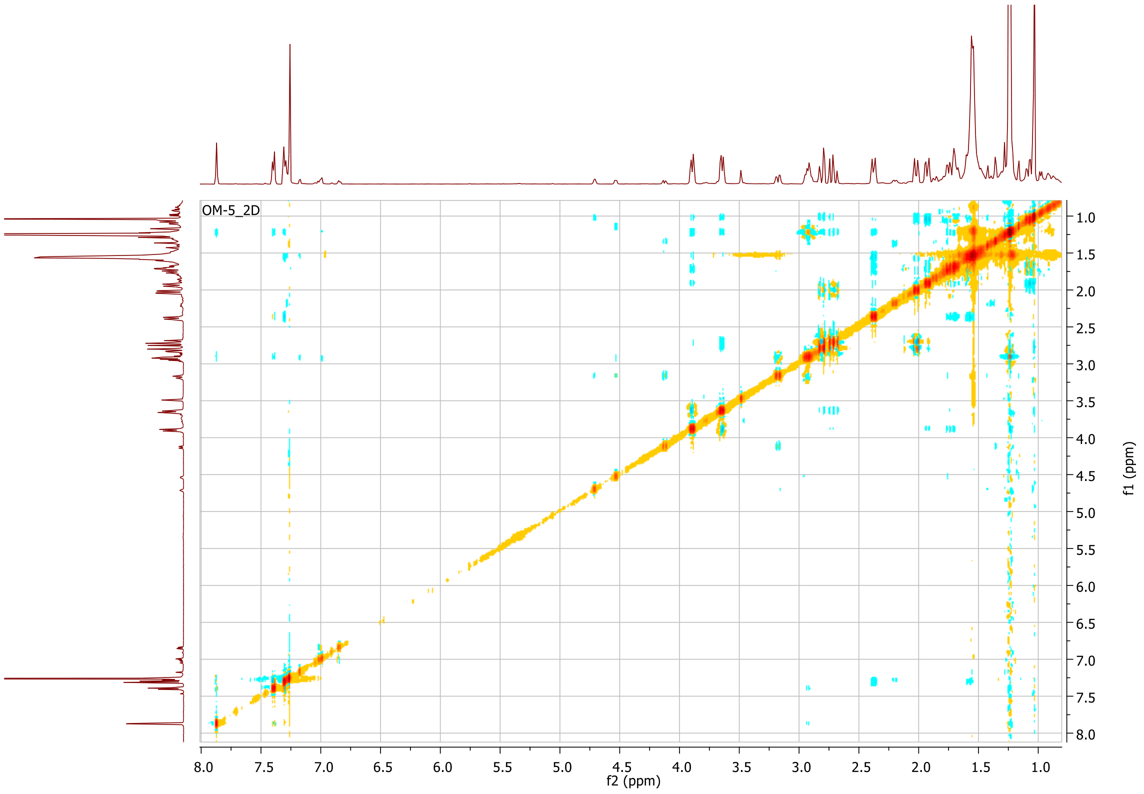

Figure 12. NOESY spectrum of compound **3** ($CDCl_3$, 500 MHz)

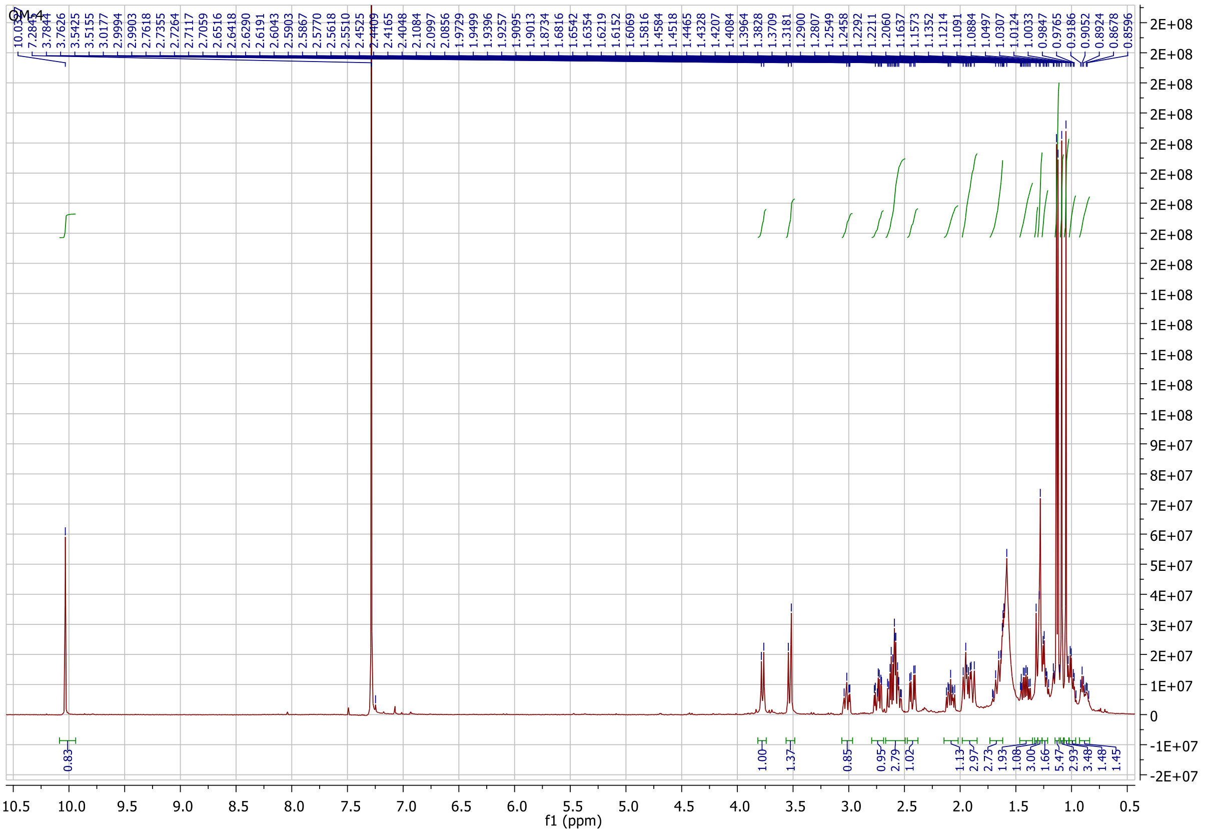

Figure 13. ^{1}H NMR spectrum of compound **4** ($CDCl_3$, 500 MHz)

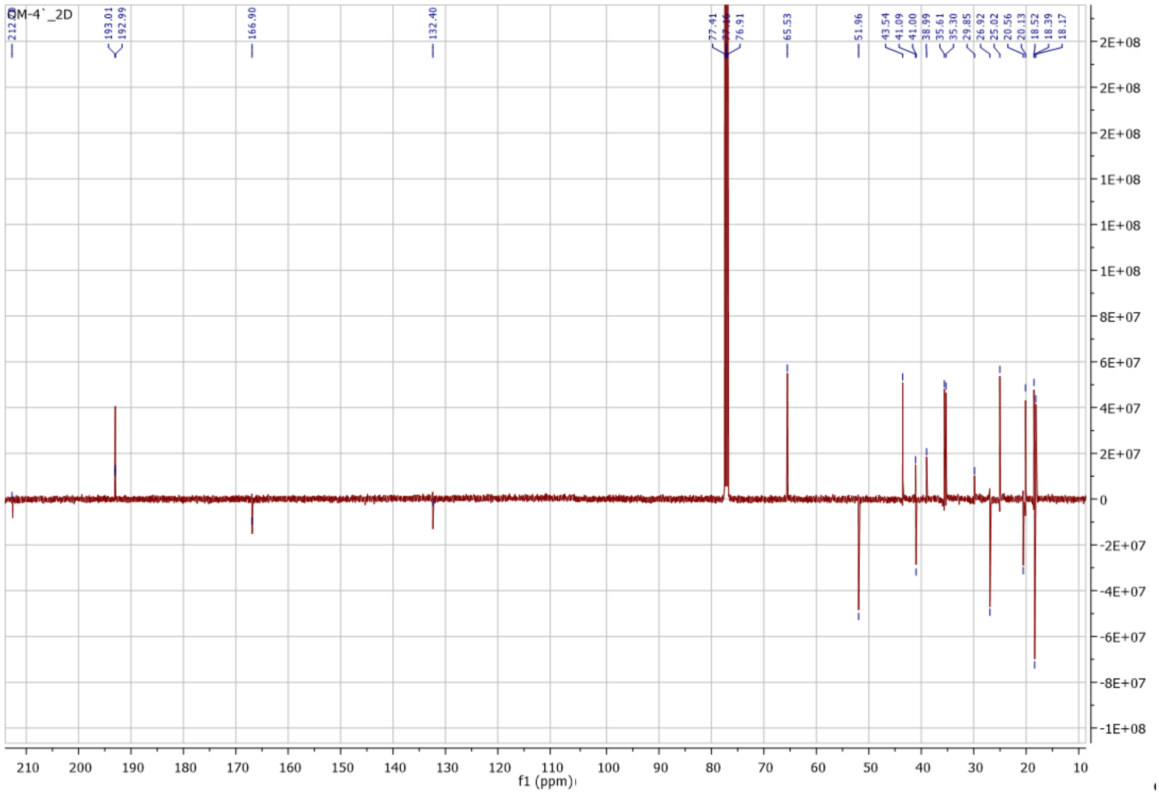

Figure 14. ^{13}C NMR JMOD spectrum of compound **4** ($CDCl_3$, 125 MHz)

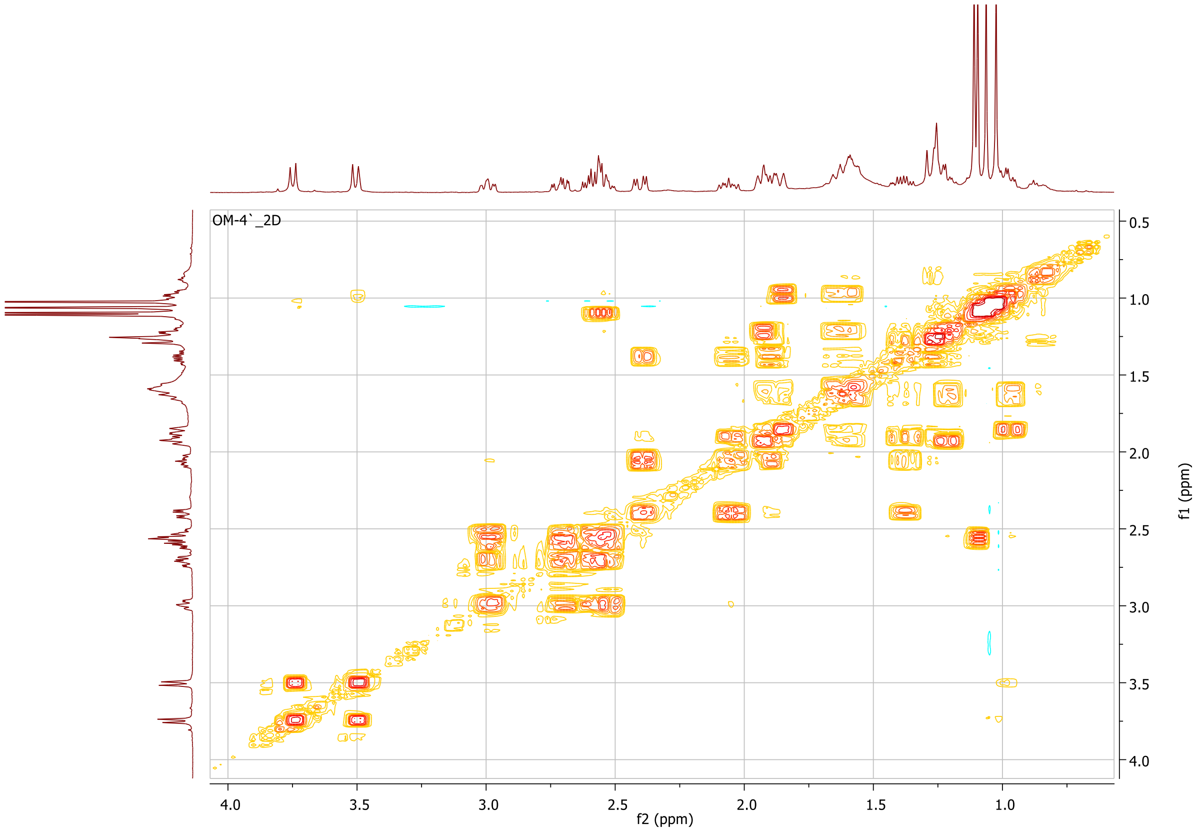

Figure 15. ^1H-^1H COSY spectrum of compound **4** ($CDCl_3$, 500 MHz)

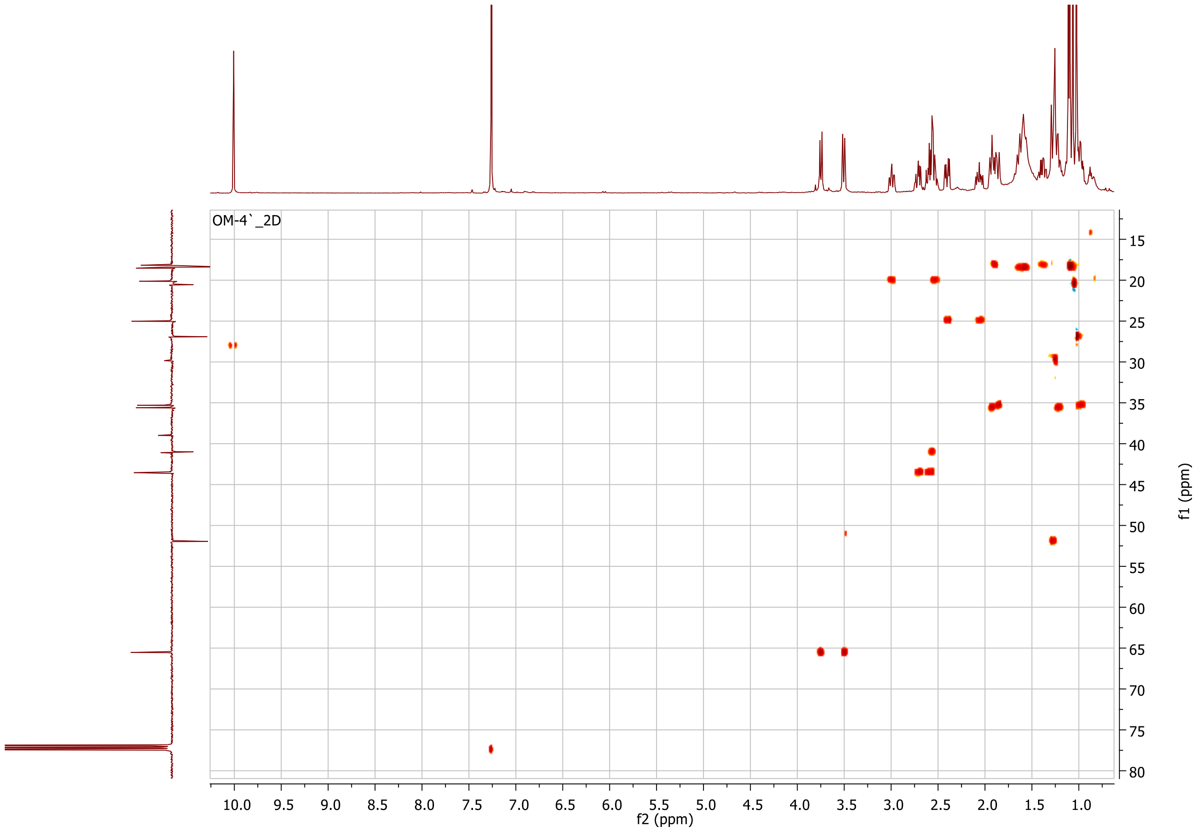

Figure 16. HSQC spectrum of compound **4** ($CDCl_3$, 125/500 MHz)

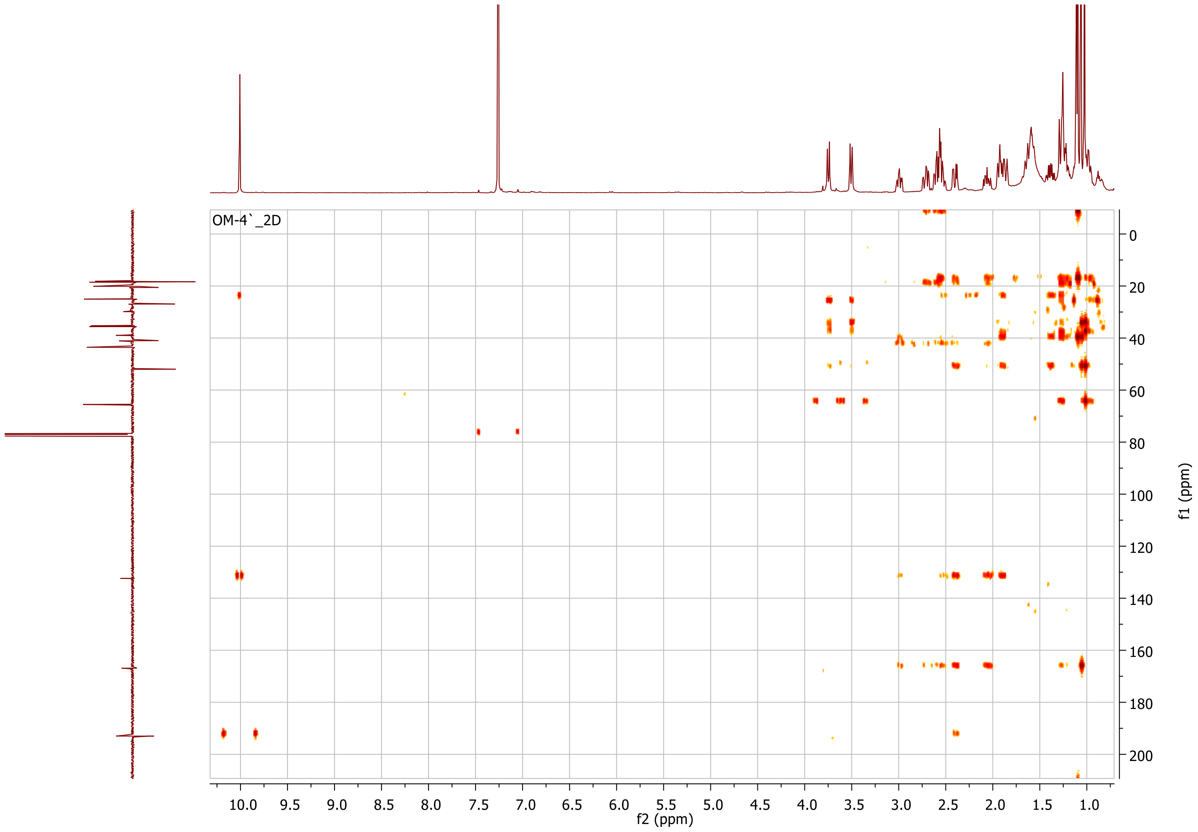

Figure 17. HMBC spectrum of compound **4** ($CDCl_3$, 125/500 MHz)

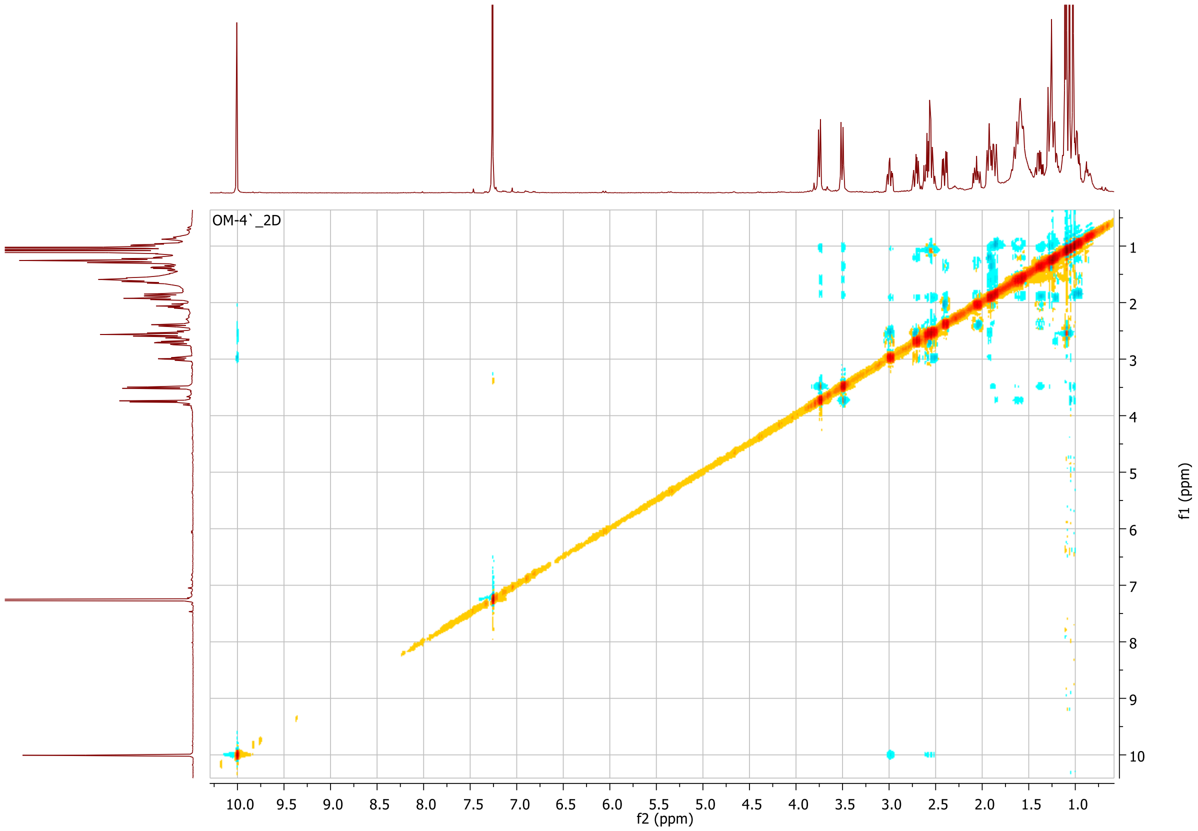

Figure 18. NOESY spectrum of compound **4** ($CDCl_3$, 500 MHz)

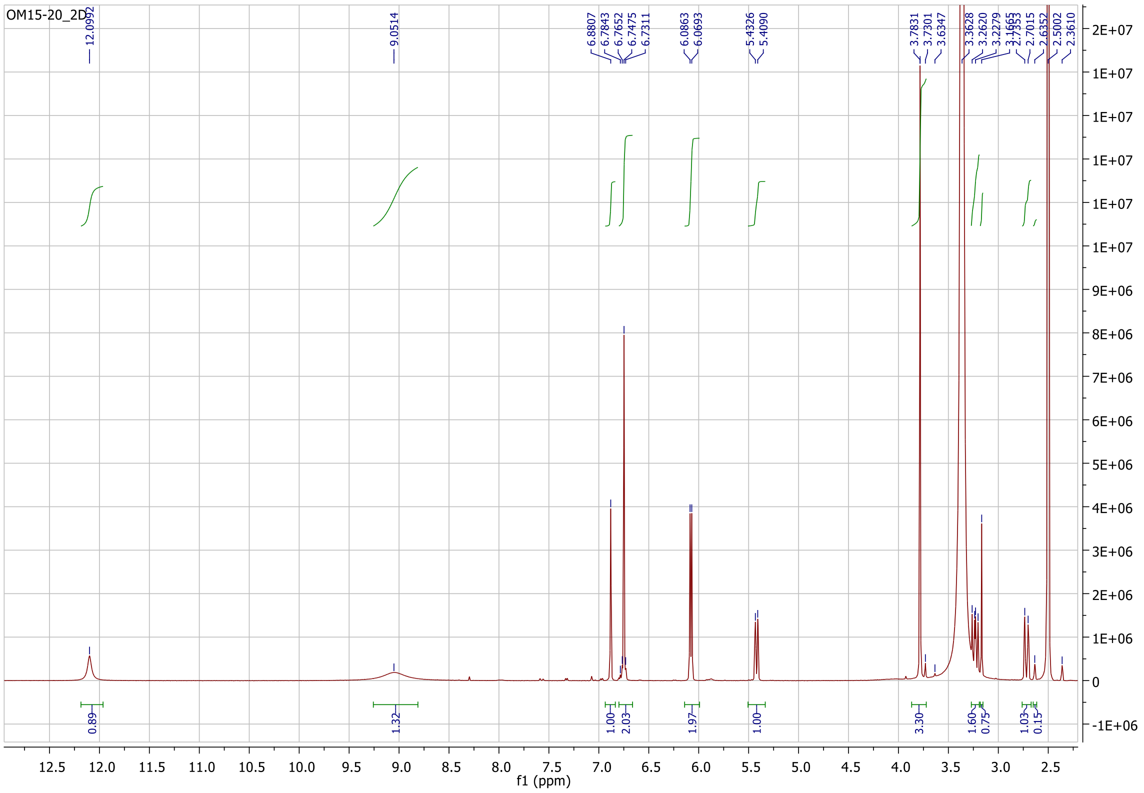

Figure 19. ^{1}H NMR spectrum of compound **5** (DMSO-d_6, 500 MHz)

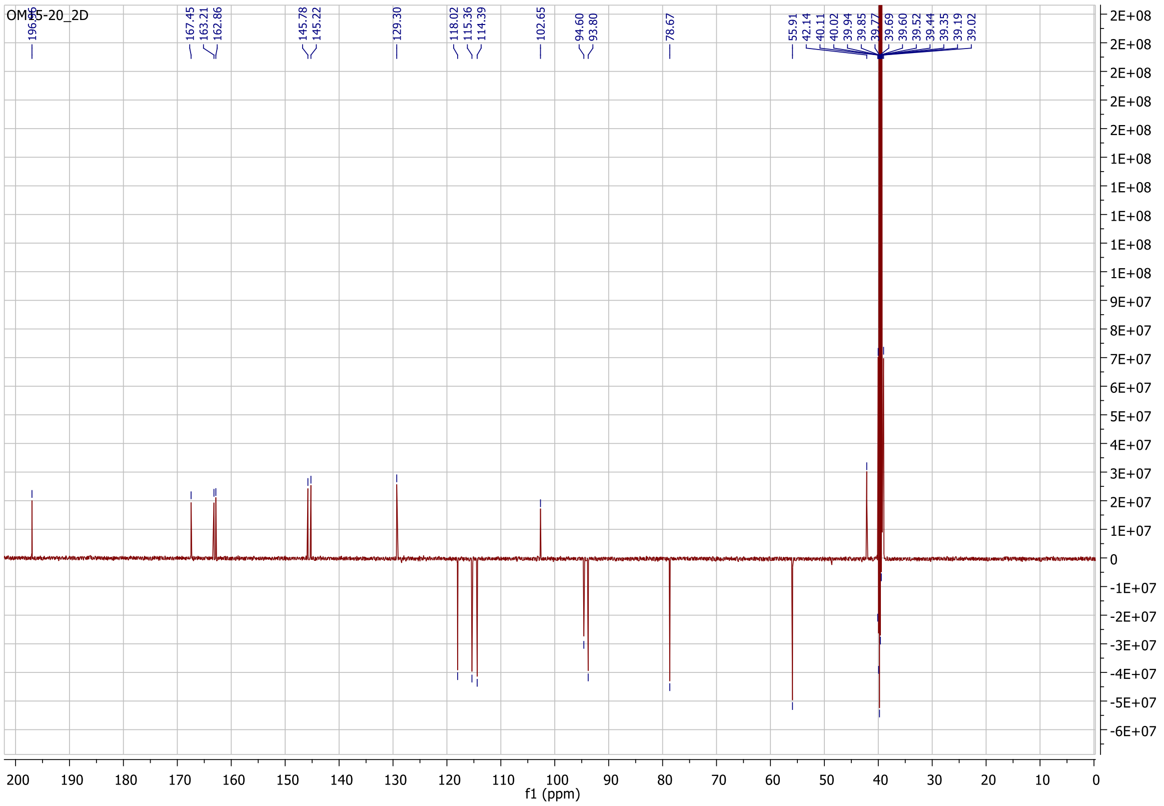

Figure 20. ^{13}C NMR JMOD spectrum of compound **5** (DMSO-d_6, 125 MHz)

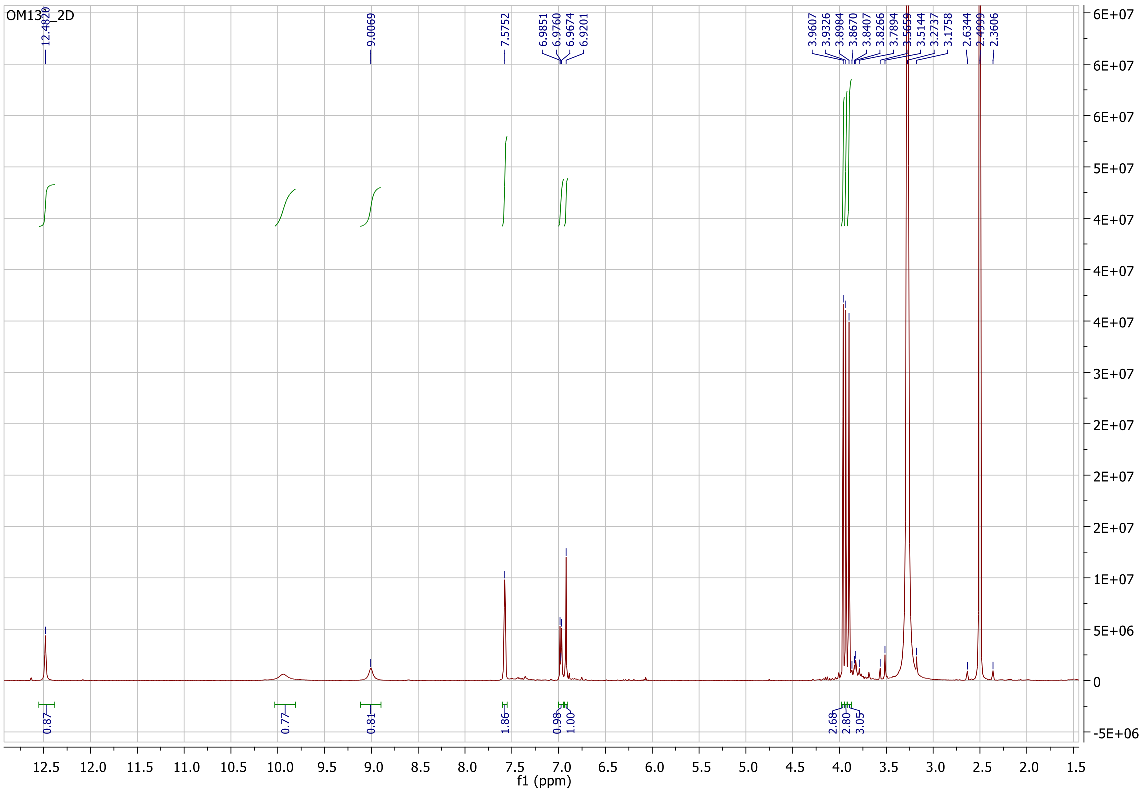

Figure 21. ^{1}H NMR spectrum of compound **6** (DMSO-d_6, 500 MHz)

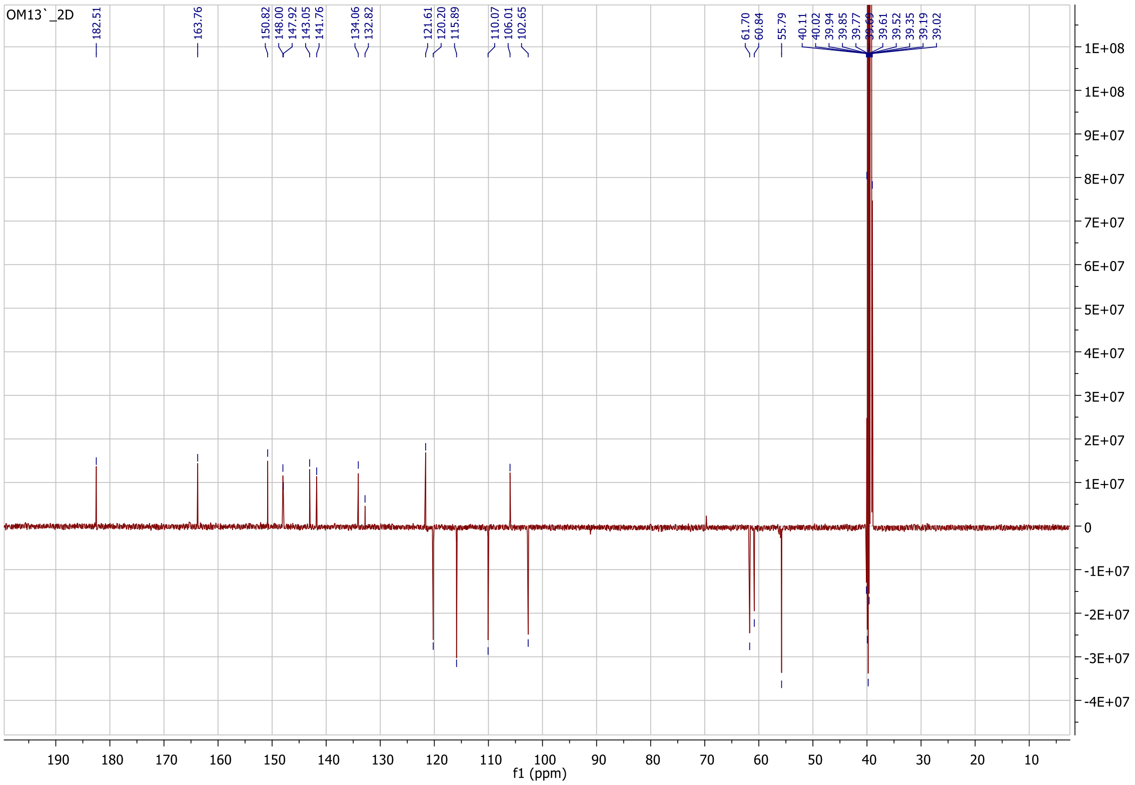

Figure 22. ^{13}C NMR JMOD spectrum of compound **6** (DMSO-d_6, 500 MHz)

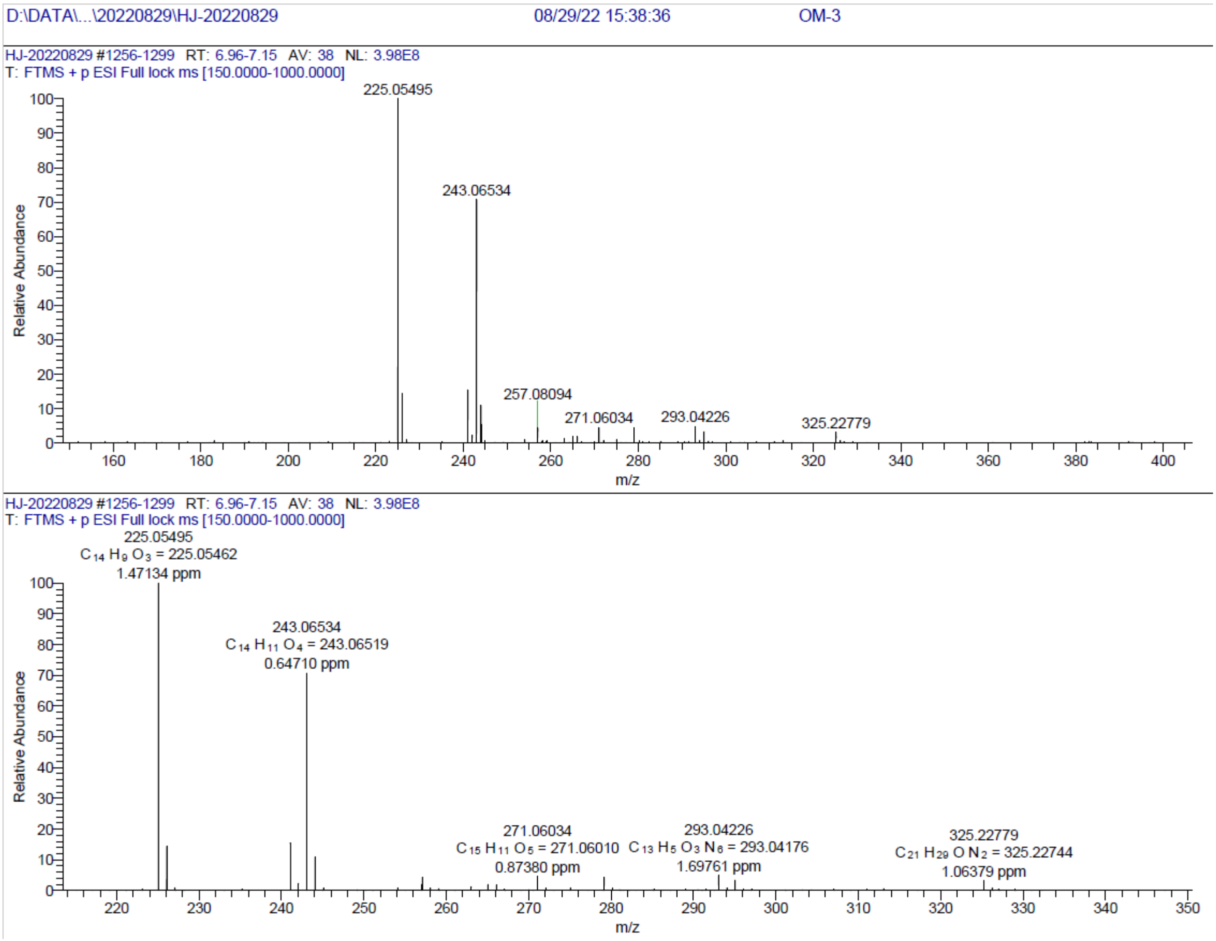

Figure 23. HRESIMS of compound **1**

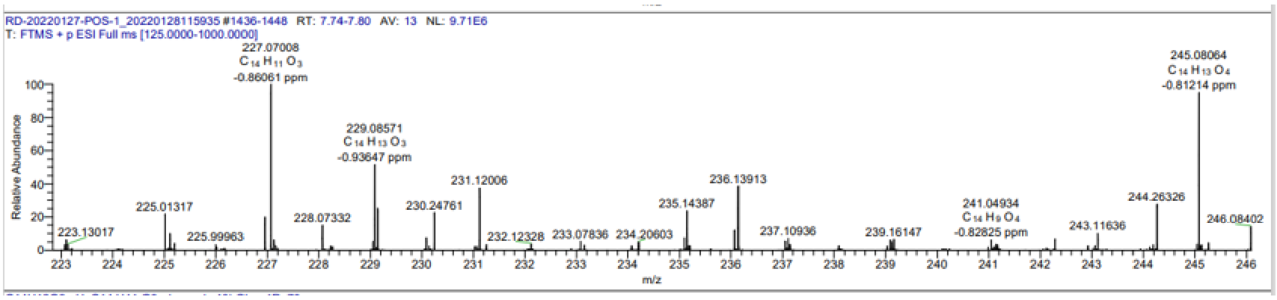

Figure 24. HRESIMS of compound **2**

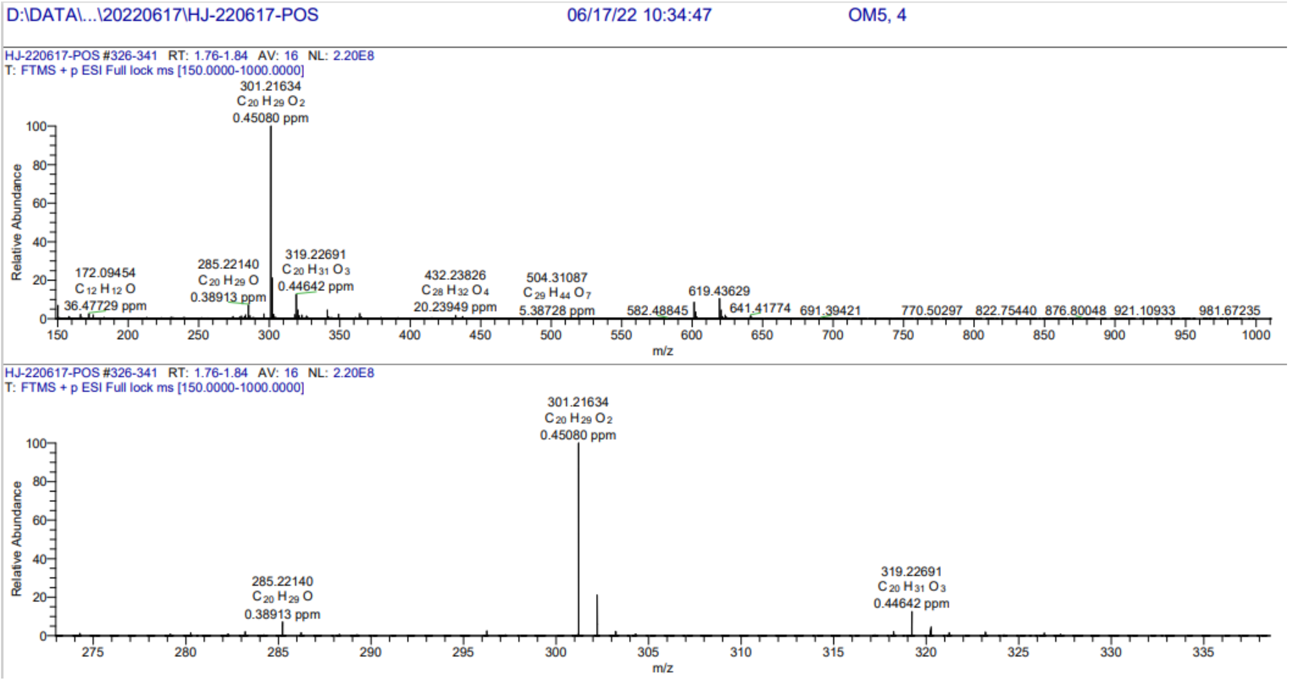

Figure 25. HRESIMS spectrum of compound **3**

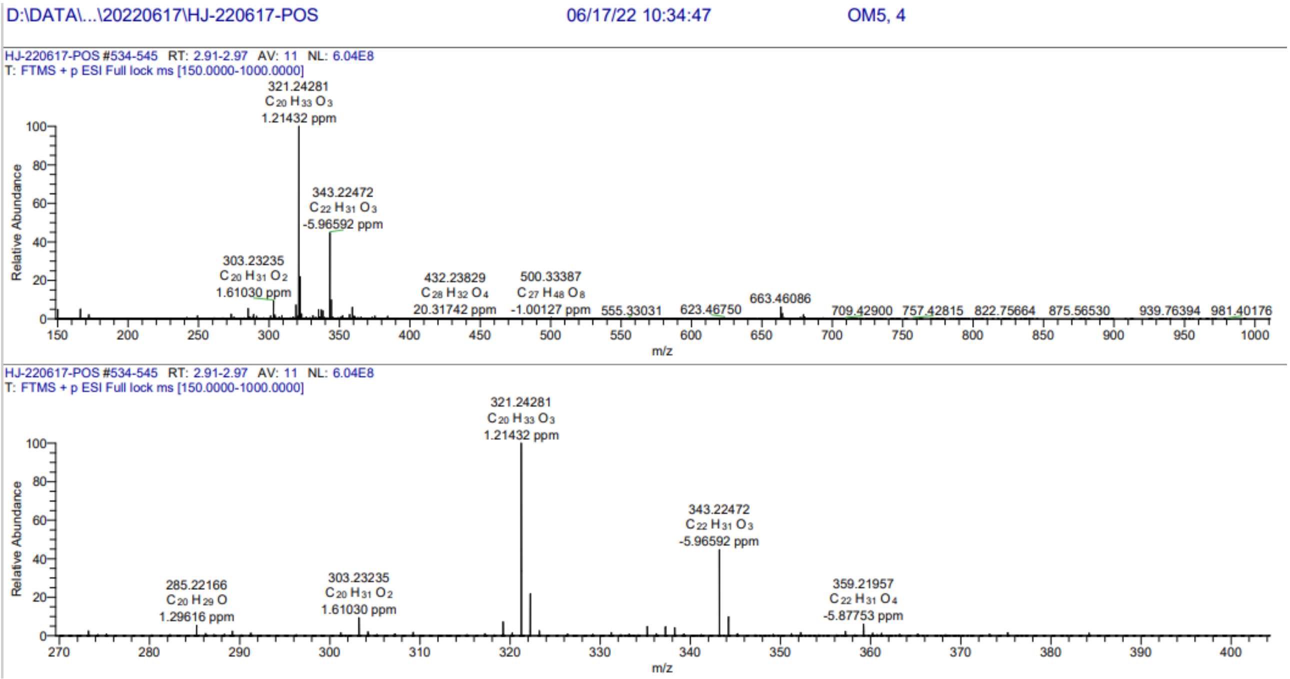

Figure 26. HRESIMS spectrum of compound **4**

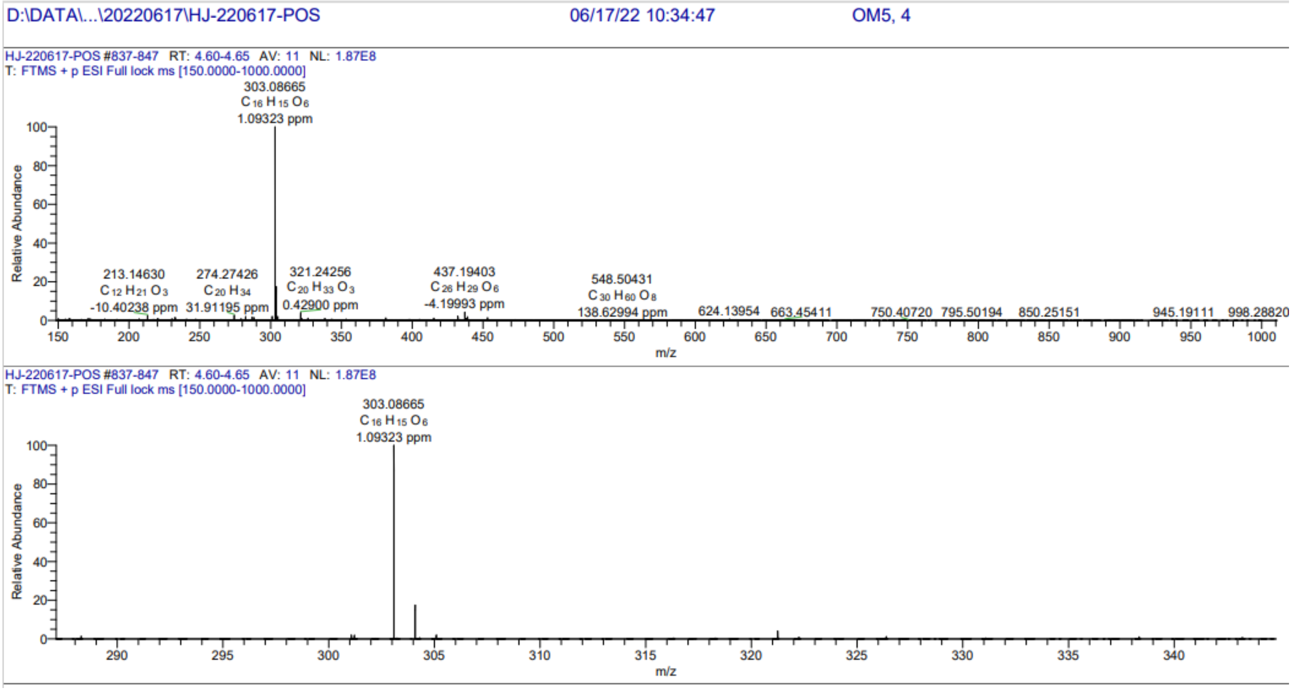

Figure 27. HRESIMS spectrum of compound **5**

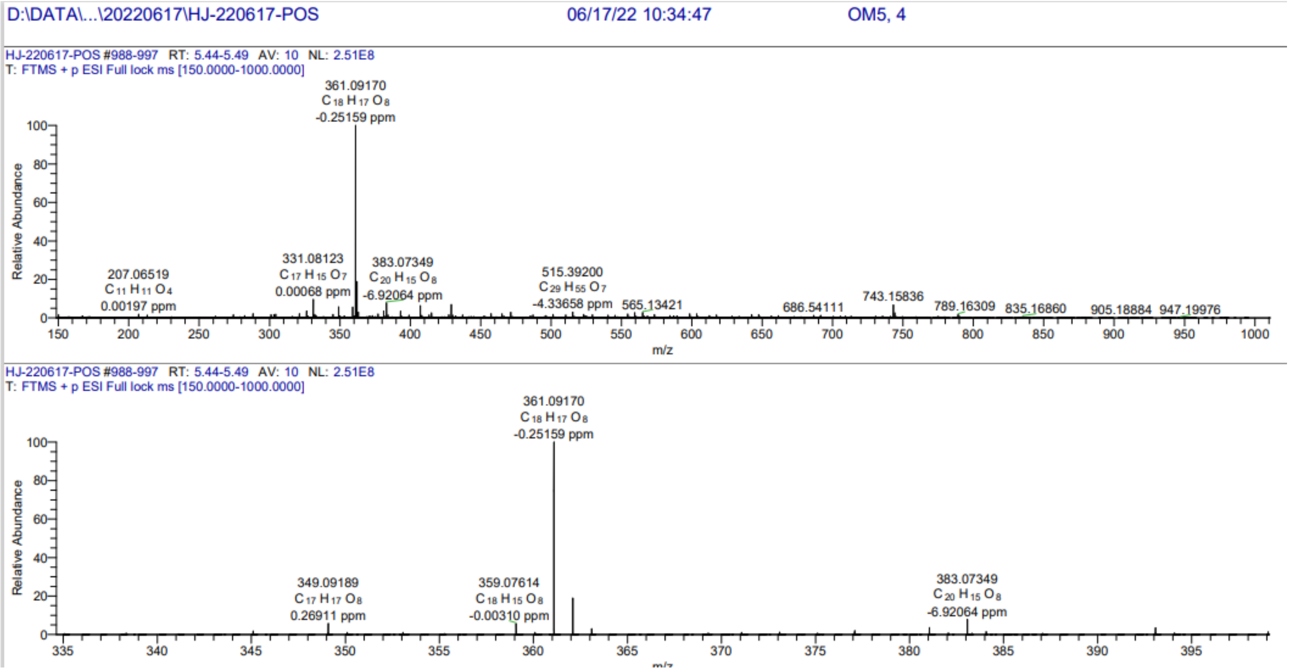

Figure 28. HRESIMS spectrum of compound **6**

Table 1. Minimum inhibitory concentration (MIC) values of majoranaquinone (**1**)

MIC (µM)

	S.aureus ATCC 25923	*S.aureus* MRSA ATCC 43300	*E.coli* ATCC 25922	*E.coli* AG100
compd 1	125	125	>1000	>1000

Compound concentration stock: 100 mM
starting concentration: 1 mM

Table 2. Relative fluorescence index (RFI) of majoranaquinone (**1**) against *E. coli* and *S. aureus* strains

		1	2	3	4	5	6	7	8	9	10	11	12	
A	*E.coli* ATCC 25922	122065	158392	150824		38289	41837	43137		42078	45605	47491		*E.coli* AG100
B		120499	145335	122351		38020	42290		39542	39038	41407		48450	
C		107548	112237	112164	107440	35541	42429	66524	68957	64778	69999	47533	48181	
D						35206						46695	44921	
E	*S.aureus* ATCC 25923	30725	31999	32120	32653	47320		51151		44117	44164	42073		MRSA ATCC 43300
F						48453						43697		
G		52716	58464	54123	52904	49228		46273	51217		45176	43355		
H		46004	43963		42690	46301		40924	44894	40941	42076	43340		

E.coli ATCC 25922

CC. (µM)	Cpd.	AV.	SD.	RFI
500	compd 1	143760,3	19165,97	2,91
1000	compd 1	129395	13835,47	2,52
50	CCCP	109847,3	2717,82	1,99
2%	DMSO	36764	1615,16	-
-	Bact.	42185,33	309,57	0,15

E.coli AG100

CC. (µM)	Cpd.	AV.	SD.	RFI
500	compd 1	43606,67	1809,80	-0,08
1000	compd 1	39995,67	1247,96	-0,15
50	CCCP	67564,5	2360,30	0,43
2%	DMSO	47239,67	472,16	-
-	Bact.	47184	1964,43	0,00

Concentration: 500 and 1000 µM, because MIC values were >1000 µM

S.aureus ATCC 25923

CC. (µM)	Cpd.	AV.	SD.	RFI
62,5	compd 1	31874,25	817,15	-0,28
25	RES	54551,75	2681,70	0,23
2%	DMSO	44219	1671,77	-
-	Bact.	47825,5	1283,28	0,08

MRSA ATCC 43300

CC. (µM)	Cpd.	AV.	SD.	RFI
62,5	compd 1	46477,33	4047,58	0,10
25	RES	47555,33	3218,18	0,13
2%	DMSO	42208,75	1869,58	-
-	Bact.	43116,25	714,77	0,02

Concentration: MIC/2

Table 3. Biofilm formation inhibitory activity of majoranaquinone (**1**) on *E. coli*

The samples are color coded using the color of the result sections below.

n/a: these data were removed in the calculations

	1	2	3	4	5	6	7	8	9	10	11	12
A	1,654	n/a	n/a	1,613	1,854	1,766	1,229	n/a	1,169	1,287	1,284	1,191
B	1,451	n/a	1,608	1,337	n/a	1,601	0,907	1,078	0,979	1,003	n/a	n/a
C	n/a	0,959	n/a	0,897	n/a	0,8417	0,996	0,989	0,814	0,973	n/a	1,005
D	1,079	1,017	n/a	1,059	n/a	0,9354	0,916	0,829	1,057	0,973	1,072	1,074
E	2,293	n/a	2,494	n/a	0,993	n/a	0,985	n/a	0,863	n/a	1,135	1,348
F	2,438	n/a	n/a	0,983	n/a	n/a	0,809	0,839	0,614	n/a	1,231	1,282
G	2,187	2,146	2,137	0,969	0,972	n/a	0,926	0,985	n/a	1,369	n/a	n/a
H	2,316	n/a	2,366	0,818	n/a	n/a	0,933	0,83	n/a	n/a	1,245	1,182
			E.coli ATCC 25922						*E.coli* AG100			

cc.	Cpd.	AV.	SD	Cpd. OD	Inhibition %
500 µM	compound 1	1,72	0,11	0,77	42,62
1000 µM	compound 1	1,50	0,13	0,55	59,10
50 µM	CCCP	0,90	0,06	-0,05	103,54
50 µg/mL	TZ	1,02	0,06	0,08	94,40

cc.	Cpd.	AV.	SD	Cpd. OD	Inhibition %
500 µM	compound 1	1,23	0,05	0,37	6,14
1000 µM	compound 1	0,99	0,07	0,13	67,56
50 µM	CCCP	0,96	0,08	0,09	76,86
50 µg/mL	TZ	0,99	0,10	0,12	68,82

Concentration: 500 and 1000 µM, because MIC values were >1000 µM

	Untreated control and medium				Biofilm %
-	*E.coli* ATCC 25922	2,30	0,13	1,35	100
-	LB	0,95	0,07	0,00	0

	Untreated control and medium				Biofilm %
-	*E.coli* AG100	1,26	0,08	0,39	100
-	LB	0,86	0,11	0,00	0

Table 4 Biofilm formation inhibitory activity of majoranaquinone (**1**) on *S. aureus*

	1	2	3	4	5	6	7	8	9	10	11	12
A	1,581	1,89	1,643	n/a	1,821	n/a	2,536	2,456	2,71	n/a	2,967	2,686
B	n/a	0,949	0,877	1,069	n/a	1,027	1,226	1,331	1,432	1,16	1,386	1,2
C	0,904	0,936	n/a	0,941	0,843	n/a	n/a	1,133	0,946	n/a	1,283	1,223
D	1,191	n/a	1,104	0,942		0,839	0,912	0,943	n/a	2,815	2,794	2,97
E	n/a	1,668	1,766	0,96	1,099	0,903	n/a	0,817	0,887	2,86	2,937	2,981
F	1,562	1,624	1,602	0,96	1,015	n/a	0,976	n/a	0,951	2,927	2,94	n/a
G	n/a	1,63	1,704	0,977	0,977	0,888	0,923	0,912	0,951	2,836	n/a	2,772
H	1,725	1,455	1,432	0,877	n/a	n/a	0,802	n/a	n/a	2,772	2,659	2,953
		***S.aureus* ATCC 25923**						***S.aureus* MRSA ATCC 43300**				

The samples are color coded using the color of the result sections below.

n/a: these data were removed in the calculations

cc.	Cpd.	AV.	SD	Cpd. OD	Inhibition %
**62.5 µM**	**compound 1**	1,73	0,15	0,78	**-33,09**
**50 µM**	**CCCP**	0,98	0,09	0,03	**94,63**
**50 µg/mL**	**TZ**	0,91	0,05	-0,04	**107,26**

cc.	Cpd.	AV.	SD	Cpd. OD	Inhibition %
**62.5 µM**	**compound 1**	2,67	0,20	1,76	**9,81**
**50 µM**	**CCCP**	1,29	0,11	0,38	**80,48**
**50 µg/mL**	**TZ**	1,15	0,15	0,24	**87,78**

Concentration: MIC/2

	Untreated control and medium				Biofilm production%
-	***S.aureus* ATCC 25923**	1,54	0,21	0,59	**100**
-	**TSB**	0,95	0,07	0,00	**0**

	Untreated control and medium				Biofilm production%
-	**MRSA ATCC 43300**	2,86	0,10	1,96	**100**
-	**TSB**	0,91	0,10	0,00	**0**
